# The Absence of a Very Long Chain Fatty Acid (VLCFA) in Lipid A Impairs *Agrobacterium fabrum* Plant Infection and Biofilm Formation and Increases Susceptibility to Environmental Stressors

**DOI:** 10.3390/molecules30051080

**Published:** 2025-02-26

**Authors:** Iwona Komaniecka, Kamil Żebracki, Andrzej Mazur, Katarzyna Suśniak, Anna Sroka-Bartnicka, Anita Swatek, Adam Choma

**Affiliations:** 1 Department of Genetics and Microbiology, Institute of Biological Sciences, Maria Curie-Sklodowska University, 20-033 Lublin, Poland; kamil.zebracki@mail.umcs.pl (K.Ż.); andrzej.mazur@mail.umcs.pl (A.M.); anita.swatek@mail.umcs.pl (A.S.); 2 Department of Pharmaceutical Microbiology, Medical University of Lublin, Chodźki 1, 20-093 Lublin, Poland; katarzyna.susniak@umlub.pl; 3 Independent Unit of Spectroscopy and Chemical Imaging, Medical University of Lublin, Chodźki 4a, 20-093 Lublin, Poland; anna.sroka-bartnicka@umlub.pl

**Keywords:** *Agrobacterium fabrum*, lipid A, very long chain fatty acids, mass spectrometry, biofilm, FTIR spectroscopy, plant infection

## Abstract

The *Agrobacterium fabrum* C58 is a phytopathogen able to infect numerous species of cultivated and ornamental plants. During infection, bacteria genetically transform plant cells and induce the formation of tumours at the site of invasion. Bacterial cell wall components play a crucial role in the infection process. Lipopolysaccharide is the main component of Gram-negative bacteria’s outer leaflet of outer membrane. Its lipophilic part, called lipid A, is built of di-glucosamine backbone substituted with a specific set of 3-hydroxyl fatty acids. *A. fabrum* incorporates a very long chain hydroxylated fatty acid (VLCFA), namely 27-hydroxyoctacosanoic acid (28:0-(27OH)), into its lipid A. *A. fabrum* C58 mutants deprived of this component due to mutation in the VLCFA’s genomic region, have been characterised. High-resolution mass spectrometry was used to establish acylation patterns in the mutant’s lipid A preparations. The physiological properties of mutants, as well as their motility, ability to biofilm formation and plant infectivity, were tested. The results obtained showed that the investigated mutants were more sensitive to environmental stress conditions, formed a weakened biofilm, exhibited impaired swimming motility and were less effective in infecting tomato seedlings compared to the wild strain.

## 1. Introduction

*Agrobacterium fabrum* C58 (also referred to as *A. tumefaciens* C58) is a Gram-negative, soil-dwelling, phytopathogenic bacterium belonging to the *Rhizobiaceae* family [1,2]. These bacteria infect a wide range of dicotyledonous plants (over 800 species) and some monocotyledons, including economically significant crops (e.g., tomatoes, grapes, plums, and apples) and ornamentals (e.g., roses) [3]. During infection, agrobacteria respond mainly to phenolic compounds synthesised by wounded plant tissues, such as acetosyringone, which are recognised by a VirA/VirG two-component regulatory system. After the recognition, the transcriptional regulator VirG is phosphorylated and activates *vir*-gene expression after binding to a *vir*-box at agrobacterial Ti plasmid [4]. Agrobacteria invade the host and genetically transform plant cells by transferring T-DNA—a fragment of agrobacterial Ti plasmid—via a type IV secretion system (T4SS). The integrated T-DNA induces the expression of genes that stimulate excessive auxin and cytokinin production, leading to crown gall tumour formation. Infected cells also produce opines, derivatives of amino acids and oxoacids, which serve as carbon and nitrogen sources for *Agrobacterium* but not for other rhizosphere bacteria. This process allows *Agrobacterium* to create a specialised microenvironment tailored to its survival [5].

The survival, virulence, motility-to-sessility transition, adhesion to plant cells, and subsequent biofilm formation in *A. fabrum* rely heavily on the structural adaptability of its cytoplasmic and outer membranes. The ability of the outer membrane to rapidly restore homeostasis under changing environmental conditions seems to be crucial for the *Agrobacterium*’s persistence in soil and colonised zones. It has been proved that many bacterial processes critically depend on the composition and spatial organisation of their membranes. In Gram-negative bacteria like *A. fabrum*, lipopolysaccharides (LPS) are essential components of the outer membrane (OM), forming the outer leaflet of the OM and consisting of three conserved domains: the O-polysaccharide chain (OPS or O-antigen), the core oligosaccharide, and lipid A. Together with phospholipids, LPS forms a highly asymmetric bilayer, commonly referred to as the OM [6,7].

Lipid A, the most conserved region of the LPS, plays a key role in determining the OM’s spatial organisation and biological activity. Its backbone typically comprises two D-glucosamine (d-GlcN) residues linked by a β-(1,6)-glycosidic bond, decorated with orthophosphate, phosphoethanolamine, or uronic acid at the C-1 and C-4′ positions [6]. This structural framework is consistent among members of the *Enterobacteriaceae* family and the *Rhizobium* genus [8]. Lipid A is acylated at the C-2, C-2′, C-3, and C-3′ positions with saturated, straight or branched, 3-hydroxy or occasionally 2-hydroxy fatty acids. The specific fatty acid composition and arrangement, referred to as the acylation pattern, are characteristic of each bacterial genus [9]. The lipid A structure of *Escherichia coli* has been extensively studied and serves as a model for comparative analyses [6].

The lipid A structure described for *A. fabrum* closely resembles the enterobacterial model, with differences primarily in the acylation pattern. Similar to most rhizobial lipid A molecules, it is penta-acylated, though the isolated lipid A pool is structurally diverse. The major fraction of lipid A molecules consists of two ester-linked 3-hydroxymyristic acid residues (14:0-(3OH)) and two amide-linked 3-hydroxypalmitic acid residues (16:0-(3OH)). At the C-2′ position of the distal d-GlcN residue, the 16:0-(3OH) may be replaced by 3-hydroxyoctadecenoic or 3-hydroxyoctadecanoic acids (18:1-(3OH) or 18:0-(3OH)), which are further modified with a very long-chain fatty acid (VLCFA), specifically 27-hydroxyoctacosanoic acid (28:0-(27OH)), forming an acyloxyacyl moiety. This structure is additionally acylated with 3-hydroxybutyrate (4:0-(3OH)) linked at the (ω−1) position. The other variants of lipid A molecules can differ by the absence of a phosphate group at the C-1 position, the lack of 14:0-(3OH) at C-3, or the absence of the tertiary substituent 4:0-(3OH) [10].

The structural integrity of the OM in Gram-negative bacteria is strictly dependent on the lipid A structure. This molecule influences the physiological properties of symbiotic bacteria, including their ability to establish close interactions with host plants [8]. Although *A. fabrum* belongs to the *Rhizobiaceae* family—predominantly composed of symbiotic bacteria—it acts as a pathogen when interacting with plants. Like rhizobia and other intracellular pathogenic bacteria such as *Brucella* and *Legionella*, *Agrobacterium* can synthesise VLCFAs and incorporate them into its lipid A. Rhizobial VLCFAs, ranging from 26 to 34 carbon atoms in length, most commonly include 28:0-(27OH) [8,11,12,13,14,15]. These specific fatty acids span the entire OM, creating a more compact structure that enhances bacterial resistance to environmental stressors.

Rhizobia, as well as *Agrobacterium* species that synthesise lipid A substituted with VLCFAs, possess a highly conserved gene cluster located between the *acpXL* and *msbB* (*lpxXL*) genes (where “XL” means “extra-long” in contrast to the normal length fatty acids associated with a basic metabolism). In various members of the *Rhizobiaceae* family, this region—responsible for the biosynthesis and transfer of VLCFA into the lipid A molecule—exhibits significant conservation in gene content and organisation [8]. The corresponding gene clusters include five putative open reading frames (ORFs) in slow-growing *Bradyrhizobium* and medium-growing *Mesorhizobium* species, six ORFs in fast-growing *Rhizobium, Sinorhizobium* and *Agrobacterium* species, and up to seven ORFs in *Azorhizobium caulinodans*. Genes within the *acpXL*–*msbB* region are always oriented in the same direction and form at least one operon. In each case, a strong promoter sequence can be predicted upstream of the *acpXL* genes [8]. In *A. fabrum*, this region includes six genes: *acpP* (acyl carrier protein), *fabZ* (3-hydroxyacyl-ACP dehydratase), *fabF* (3-oxoacyl-ACP synthase II), *fabF* (3-oxoacyl-ACP synthase I), *Atu1595* (alcohol dehydrogenase), and *msbB* (acyltransferase).

The lipid A structures of *Agrobacterium* and *Sinorhizobium* show a high degree of similarity [10,16]. Moreover, the *acpXL*–*msbB* region of *S. meliloti* has been fully characterised, including the generation of mutants for all genes within the VLCFA locus and detailed analyses of their physiological and symbiotic properties [16,17]. The gene content of the *acpXL*–*msbB* region in *A. fabrum* closely resembles that in *S. meliloti*, suggesting that the corresponding genes in *Agrobacterium* likely encode proteins with similar functions. Although the roles of individual genes within the *acpXL*–*msbB* cluster of *A. fabrum* have been predicted, the genetic organisation of the *acpXL*–*msbB* gene cluster and how the inactivation of these genes (the resulting loss of VLCFA in lipid A) affects the infectious capacity of this phytopathogen remains unexplored.

The present study investigates the structural characteristics of lipid A (especially lipids A from obtained mutant) and explores the biological and physicochemical properties of mutants deficient in genes responsible for VLCFA biosynthesis. We also incorporate the genetic organisation of the *acpXL*–*msbB* gene cluster of *A. fabrum* into our investigations. These findings aim to elucidate the role of VLCFAs in the virulence and environmental adaptability of *A. fabrum*.

## 2. Results

To determine whether *A. fabrum* C58, lacking the ability to synthesise VLCFAs, retains the capacity to infect and transform dicotyledonous plants, mutants in the *fabF2XL* and *adhlA2XL* genes were constructed. Since these genes are part of a putative operon encoding enzymes in the biosynthetic pathway of VLCFAs, the mutant construction was preceded by an analysis of the transcriptional organisation of the *acpXL*–*msbB* gene cluster. The mutants were tested for their plant infection properties and response to various environmental stressors.

### 2.1. Genetic Characterisation of A. fabrum C58 acpXL–lpxXL Gene Cluster

#### 2.1.1. The acpXL–lpxXL Gene Cluster Is Organised into Two Transcriptional Units

**Figure 1:**
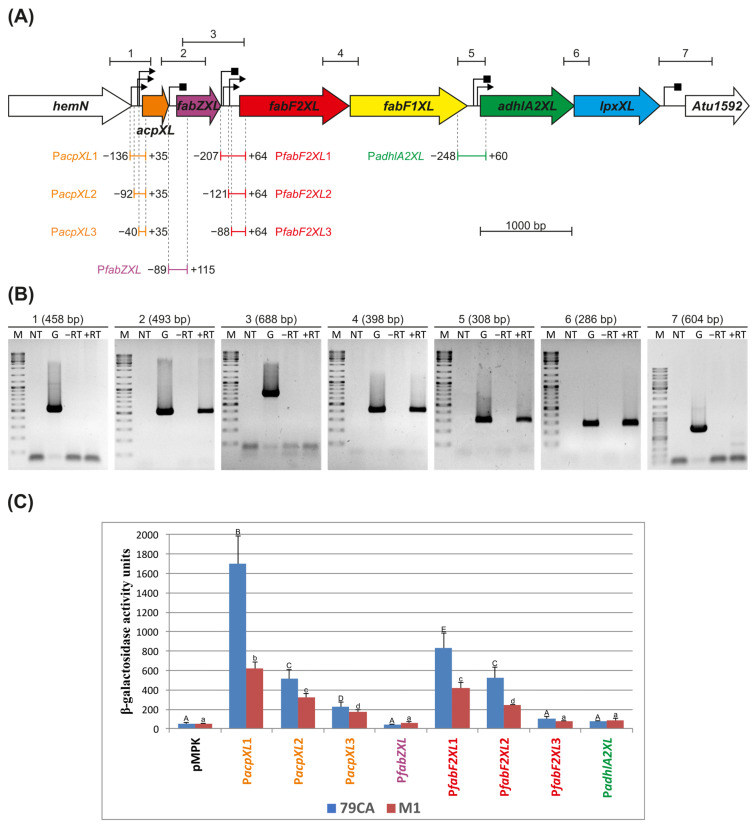
Transcriptional organisation of the *acpXL*–*lpxXL* region responsible for the biosynthesis of very long-chain fatty acids in *A. fabrum* C58 lipopolysaccharide. (**A**) Schematic representation of the 8 kbp DNA fragment encompassing the *acpXL*–*lpxXL* gene cluster. Individual open reading frames are depicted as arrows in different colours, representing their putative functions (provided in brackets): orange—*acpXL* (acyl carrier protein), purple—*fabZXL* ((3R)-hydroxyacyl-ACP dehydratase), red—*fabF2XL* (3-oxoacyl-ACP synthase II), yellow—*fabF1XL* (3-oxoacyl-ACP synthase II), green—*adhlA2XL* (dehydrogenase), and blue—*lpxXL* (lipid A biosynthesis lauroyl acyltransferase). White arrows indicate flanking genes of the *A. fabrum* C58 *acpXL*–*lpxXL* cluster. The upper part of the panel illustrates the PCR-amplified fragments along with in silico predicted promoters (black triangles) and terminators (black squares). The respective DNA fragments used to determine the transcriptional organisation and to map individual promoters are shown as coloured segments, with positions marked relative to the ATG codon of the preceding gene below the arrows representing corresponding genes. (**B**) Results of PCR reactions with primers spanning the intergenic regions of the studied gene cluster. The numbers correspond to the sections marked in panel (**A**). The sizes of the amplified DNA fragments are indicated in parentheses. Symbols used: ‘M’—DNA molecular weight marker; ‘NT’—control reaction without template DNA; ‘G’—control reaction with *A. fabrum* C58 genomic DNA; ‘−RT’—control reaction with isolated total RNA without reverse transcription; ‘+RT’—reaction with cDNA as the template. (**C**) Transcriptional activities of the hypothetical promoters in the *acpXL*–*lpxXL* region. PCR-amplified DNA fragments containing the predicted promoters upstream of selected genes were cloned into the pMPK reporter vector. The β-galactosidase activities of the transcriptional fusions were measured and expressed in Miller units. *A. fabrum* C58 cells carrying the empty pMPK vector served as a control. The presented results (with standard deviations) are the mean values from four independent experiments. Assays were conducted in 79CA (blue bars) and M1 minimal (red bars) media. Different letters indicate significant differences between groups (capital letters for measurements in 79CA medium and lowercase letters for M1 minimal medium; *p* < 0.001 for both media).

The transcriptional organisation of the *acpXL*–*lpxXL* gene cluster in *A. fabrum* C58 was investigated using a combination of in silico predictions, RT-PCR analysis, and functional promoter activity assays. This region consists of six ORFs oriented in the same direction on the *A. fabrum* chromosome (GenBank Accession: AE007869.2; Figure 1A). The partial overlap of *adhlA2XL* and *lpxXL* (4 bp), as well as the short intergenic spacing between *fabF2XL* and *fabF1XL* (12 bp), suggested common transcription for at least some genes within this cluster. The computational predictions indicated the presence of sequences that could act as promoters, which were mapped upstream of the *acpXL*, *fabF2XL*, and *adhlA2XL* genes (Supplementary Table S1), supporting the hypothesis that this cluster is organised into multiple transcriptional units. Only motifs with the highest probability of functioning as promoters (with *p* > 50% in Neural Network Promoter Prediction and a significant hit in PromoterHunter) are marked in Figure 1A. Putative intrinsic Rho-independent terminators were identified downstream of *acpXL*, *fabZXL*, *fabF1XL*, and *lpxXL* (Figure 1A; Supplementary Table S2). Conversely, the analysis of the region using the Operon-mapper tool, which relies on gene conservation and genome architecture, predicted that these genes are organised as a single operon, highlighting potential discrepancies between computational predictions and the need for experimental validation.

To experimentally verify the transcriptional organisation of this region, high-quality total RNA (free of genomic DNA) was isolated from *A. fabrum* C58 cells following a previously established protocol [18,19]. cDNA was synthesised by reverse transcription using random primers and subsequently used as a template in a series of PCR reactions with primers specific for adjacent genes (Supplementary Table S3). The observed amplicon profile (Figure 1B) confirmed the co-transcription of *acpXL* and *fabZXL* as part of a single operon, while *fabF2XL*, *fabF1XL*, *adhlA2XL*, and *lpxXL* were shown to form a second transcriptional unit. No amplification was observed between *fabZXL* and *fabF2XL*, indicating a transcriptional boundary between these two regions. The experimental data also confirmed that the *hemN* and *Atu1592* genes are not co-transcribed with the *acpXL*–*lpxXL* gene cluster (Figure 1B).

The activity of the hypothetical promoters was further examined using transcriptional fusions with the *lacZ* reporter gene. Initially, four DNA fragments encompassing the upstream regions of *acpXL*, *fabZXL*, *fabF2XL*, and *adhlA2XL* were amplified and cloned upstream of a promoterless *lacZ* gene into the pMPK reporter vector (Figure 1A). The resulting plasmids (Supplementary Table S4) were introduced into the wild-type *A. fabrum* C58 strain, and β-galactosidase activity was measured (Figure 1C). The promoter activity assays demonstrated that strong promoters precede the *acpXL* and *fabF2XL* genes, consistent with the organisation of two distinct operons within the cluster. The promoter upstream of *acpXL* (P*acpXL*) exhibited significantly higher activity than that upstream of *fabF2XL* (P*fabF2XL*) in both media tested. Promoter mapping was then attempted by constructing a series of reporter plasmids with 5′-end deletions of the *acpXL* and *fabF2XL* promoter regions (P*acpXL*1–3 and P*fabF2XL*1–3; Figure 1A). The β-galactosidase activity of these deletion derivatives suggested that the minimal promoter regions required for proper transcription initiation are located approximately −40 bp upstream of *acpXL* and −121 bp upstream of *fabF2XL*. However, additional upstream elements may be essential for full promoter activity (Figure 1C). Although the presence of multiple promoter sites in these regions cannot be excluded, further investigations are required to confirm this hypothesis. The promoter activity was influenced by the composition of the growth media, with higher activity observed in the 79CA medium compared to the M1 minimal medium. However, the relative activity of the 5′-end deletion derivatives remained consistent across the media (Figure 1C).

Collectively, these results demonstrate that the *acpXL*–*lpxXL* gene cluster in *A. fabrum* C58 is organised into two transcriptional units: *acpXL*–*fabZXL* and *fabF2XL*–*fabF1XL*–*adhlA2XL*–*lpxXL*. This bipartite organisation reflects a distinct transcriptional strategy compared to related bacteria and provides insights into the potential regulatory mechanisms controlling the expression of genes involved in VLCFA biosynthesis.

#### 2.1.2. Genetic Characterisation of fabF2 and adhA2XL *A. fabrum* C58 Mutants

To determine whether *A. fabrum* C58 *fabF2XL* and *adhlA2XL* are involved in the biosynthesis of VLCFA-modified lipid A, insertional mutants were generated for these genes (Supplementary Figures S1 and S2). The mutants, C58Δfab and C58Δadhl, were complemented by introducing plasmids carrying the respective wild-type gene sequences under the control of the P*lac* promoter (pBKfabF2XL and pBKadhlA2XL; Supplementary Table S4). PCR confirmed the presence of these plasmids in the complemented strains, C58Δfab-C and C58Δadhl-C. Lipid A was then isolated from exponential-phase cultures of the mutants and their complemented derivatives to investigate the effects of the genetic modifications.

### 2.2. Lipids A of the fabF2XL and adhA2XL Mutants Are Deprived of VLCFAs

**Figure 2:**
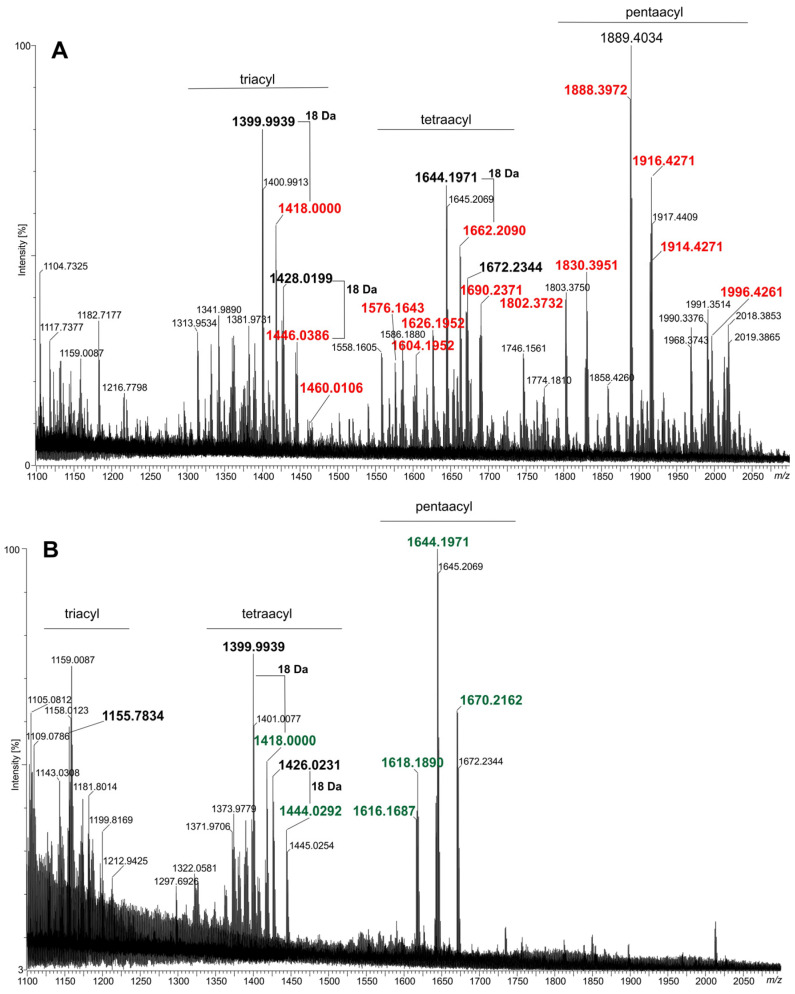

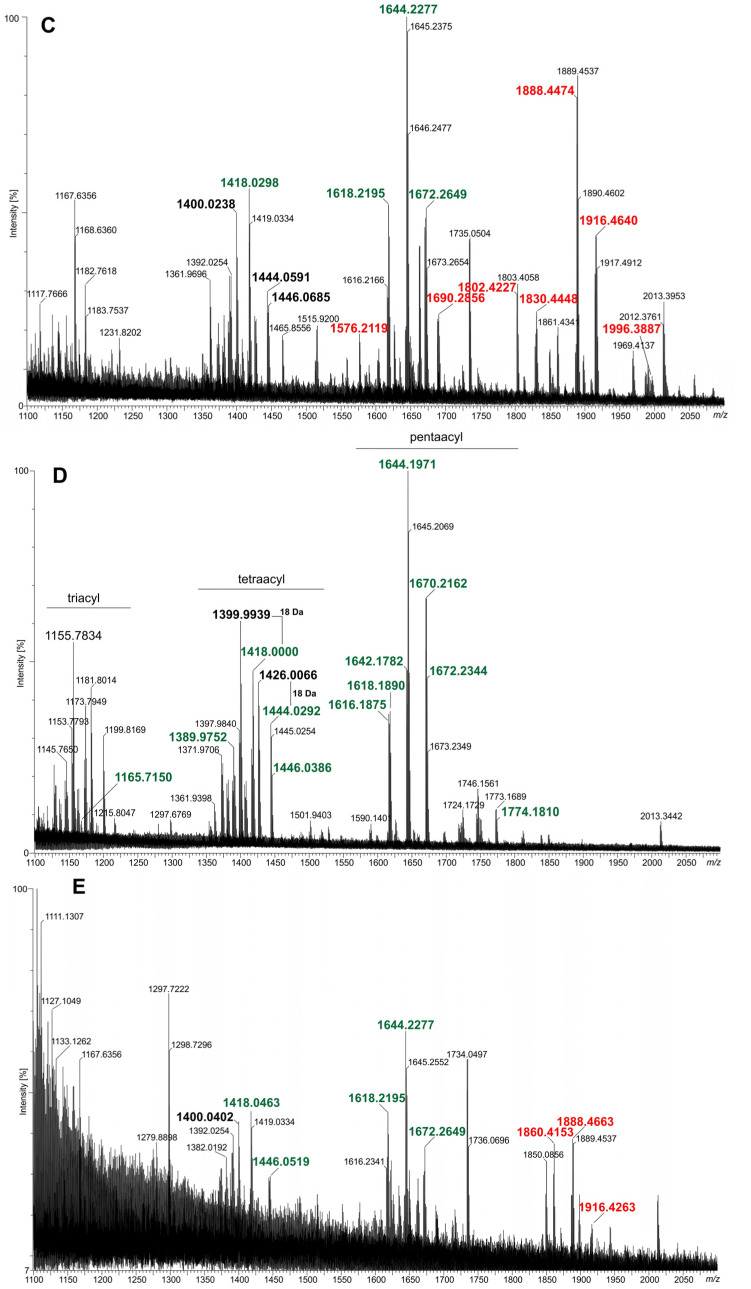
Fragments of the MALDI-TOF mass spectra (in negative ion mode) of lipid A from *A. fabrum* C58 and its derivatives: (**A**) C58Wt, (**B**) C58Δfab mutant, (**C**) C58Δfab-C (complemented strain), (**D**) C58Δadhl mutant, and (**E**) C58Δadhl-C (complemented strain). The *m*/*z* values **bolded** and marked in **red** represent ions derived from lipid A species containing VLCFAs, *m*/*z* in **green**—represent ions derived from lipid A species lacking VLCFAs, and *m/z* in **black**—represent fragment ions generated in the ion source due to the loss of water molecules or fatty acids from native lipid A species.

The only sugar identified in the lipid A preparations (obtained from the wild-type strain, mutants, and complemented strains) was glucosamine (d-GlcN). The major fatty acids detected in the lipid A of the wild-type *A. fabrum* C58 strain (C58Wt) are listed and bolded in Table 1. The GC-MS spectrum of this preparation revealed the presence of C14:0-(3OH), C16:0-(3OH), C18:0-(3OH), a small amount of C18:1-(3OH), and two VLCFAs: major C28:0-(27OH) and a minor amount of accompanying C30:0-(29OH). These fatty acid profiles, along with the presence of d-GlcN, are consistent with the previously described structure of *A. tumefaciens* C58 lipid A by Silipo and co-workers [9]. MALDI-TOF mass spectrometry analysis (Figure 2A and Supplementary Table S5) further confirmed the structure of lipid A and identified several of its molecular species in C58Wt. The primary species (pentaacylated) bears two 14:0-(3OH) fatty acids with free hydroxyl groups in an ester linkage and a pair of 16:0-(3OH), 16:0-(3OH)/18:1-(3OH), or 16:0-(3OH)/18:0-(3OH) pairs in an amide linkage. This species includes VLCFA, either 28:0-(27OH) or 30:0-(29OH), linked to the 16:0-(3OH) on the distal GlcN residue. It is decorated (but not universally) with a butyric (4:0-(3OH)) residue linked to the hydroxyl group of the VLCFA. The second species (tetraacylated), which is present in lower quantities, lacks the 14:0-(3OH) residue on the proximal GlcN. A triacylated species, present in trace amounts, lacks both ester-linked 14:0-(3OH) residues. Most of the signals in the mass spectrum (Figure 2A) of the C58Wt lipid A in the region of the so-called triacyl-molecules are generated in the spectrometer’s ion source due to the elimination of 3-OH acids from the tetraacylated lipid A form. These main species are parents for additional derivatives which lack phosphate at position C-1, 3-OH-butyrate, or both (Supplementary Table S5).

Small amounts of neutral and saturated/unsaturated fatty acids can be explained by phospholipid contamination. In the case of the mutants, the presence of these acids is probably the result of incorporating the fatty acids of the basic metabolism of the bacteria instead of the non-synthesised VLCFA.

When compared to other bacteria, it is evident that very similar lipids A are also synthesised by symbiotic bacteria such as *Sinorhizobium fredii* NGR234 (formerly *Sinorhizobium sp.* NGR234) [20] and *S. meliloti* 1021 [21]. These structural similarities are reflected in the organisation of the genetic regions encoding the biosynthetic pathways for these components of LPS [8]. Therefore, the inactivation of any gene within the *acpXL*–*lpxXL* (*msbB*) region can result in significant alterations to the structure of lipid A and LPS, leading to profound changes in the biological properties of the resulting mutants of *A. fabrum* C58. To confirm that lipid A from the C58Δfab and C58Δadhl mutants is entirely devoid of VLCFAs, the lipid A fatty acids were converted to their trimethylsilyl ether methyl esters and analysed using GC-MS (Table 1). The results demonstrated that neither mutant contained any VLCFAs in their lipid A. Notably, lipid A from the mutants showed an increased proportion of neutral fatty acids, particularly 18:1, which may reflect a compensatory mechanism. This phenomenon, where VLCFA biosynthesis deficiencies are compensated by fatty acids from the basic fatty acid synthesis pathway, has been documented in studies on rhizobial LPSs [16,22,23]. Further lipid A analyses of the complemented derivatives, C58Δfab-C and C58Δadhl-C, revealed partial restoration of VLCFA incorporation into LPS (Table 1). In C58Δadhl-C, the major VLCFA was identical to that of the wild-type strain. However, instead of the minor VLCFA 30:0-(29OH) observed in the wild-type, a shorter VLCFA was detected (26:0-(25OH).

The analysis of the lipid A preparations isolated from the C58Δfab and C58Δadhl mutant strains by MALDI-TOF MS revealed the presence of at least two major groups of lipid A species (Figure 2B,D). The major lipid A species ([M-H]^-^ type) between ions at *m*/*z* 1616 u and 1672 u, which have five acyl moieties, are listed in Supplementary Table S5. The chemical structures of three selected species of lipid A ions from this group illustrate the chemical formulas given in Supplementary Figure S3 (ions at about *m*/*z*: 1618.21 u, 1644.22 u, and 1670.21 u, respectively). The second cluster of ions (*m*/*z* values between 1370 u and 1450 u) represents lipid A molecules substituted with four fatty acids, three of which (all 3-OH fatty acids) are directly attached to the lipid A backbone. The proximal glucosamine residue contains a free OH group at the C-3 position. A neutral fatty acid is a substituent of the 3-OH group in the amide-linked acid to the distal GlcN (resulting in pentaacylated lipid A). The third cluster (around ion *m/z* at 1155 u) contained mainly fragment ions (Supplementary Figure S3, two chemical formulas on the right), and native ions (triacylated lipid A, e.g., *m*/*z* at 1165.71 u) are in a definite minority.

The replacement of eliminated VLCFAs (as a result of the inactivation of individual genes in the *acpXL*–*msbB* cluster) with simple fatty acids from basic metabolic pathways in *A. fabrum* is consistent with observations in other symbiotic bacteria [8,16,22,23]. In the MALDI-TOF MS spectra of lipids A from the complemented strains (C58Δadhl-C and C58Δfab-C, Figure 2C,E, respectively), the observed ions represent a mixture of lipid A species characteristic of the wild type and the mutants. These spectra clearly indicate partial complementation of the defects in the *fabF2XL* and *adhlA2XL* genes.

The observed partial complementation of the mutant phenotype could be explained by a high complexity of VLCFA biosynthesis pathways in rhizobia, where multiple regulatory factors influence the process. The pathway involves multiple enzymes that function in a coordinated manner, and gene expression in the complemented derivative may not match the native gene’s expression profile in the wild-type strain. This is particularly relevant given the operonic organisation of genes involved in VLCFA synthesis, as previously discussed [8]. The introduction of the gene under the control of the P*lac* promoter likely alters gene dosage, transcript stability, and protein levels, which can affect enzyme stoichiometry and activity in the VLCFA elongation process. Furthermore, VLCFA biosynthesis involves multiple iterative elongation cycles, leading to the incorporation of a very-long-chain (ω−1) hydroxylated 28:0 fatty acid into lipid A, as previously described [8]. The final steps of the process require an acyltransferase (LpxXL/MsbB) for the transfer of VLCFA to the lipid A backbone, and efficient incorporation depends on proper substrate availability, enzyme-substrate interaction, and metabolic flux. In complemented strains, discrepancies in enzyme ratios or substrate availability may reduce the efficiency of the elongation cycles or acylation steps, resulting in only partial restoration of VLCFA-modified lipid A.

### 2.3. C58Δfab Mutant LPS Displays an Altered SDS-PAGE Profile

**Figure 3:**
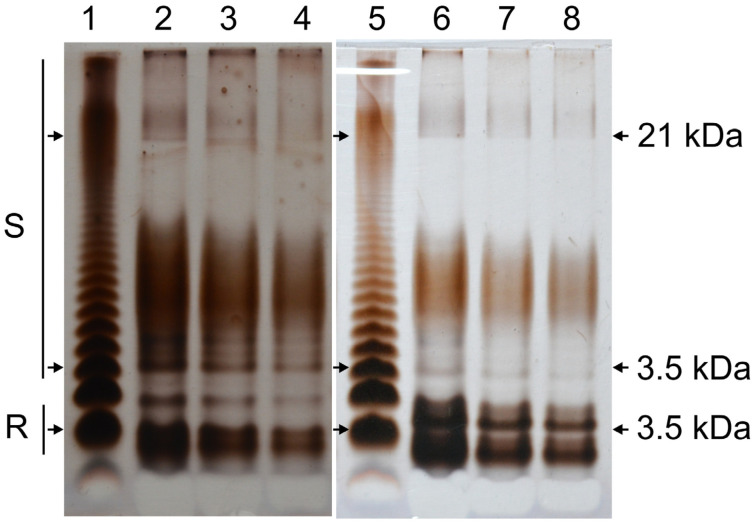
SDS-PAGE *A. fabrum* (line 2 C58Wt −4 µg; line 3 C58Wt −2 µg; line 4 C58Wt −1 µg; line 6 C58Δfab −4 µg; line 7 C58Δfab −2 µg; line 8 C58Δfab −1 µg) and *Salmonella enterica* bv. Typhimurium (lines 1 and 5, 2 µg in each line) was included as a standard for comparison of molecular weight of LPS subfractions. R—a rough type of LPS, S—a smooth type of LPS. The numbers on the right show approximated masses of the fractions marked with arrows.

The LPS from the wild-type strain and the C58Δfab mutant were analysed by SDS-PAGE (Figure 3). The LPS profile of the C58Wt strain exhibited a characteristic ladder pattern, indicative of LPS molecules with a variable number of repeating units in the O-specific polysaccharide (OPS) (lanes 2–4). In addition to the smooth (S-LPS) and rough (R-LPS) fractions, a distinct intermediate band corresponding to the semi-rough (SR) form of LPS was observed. Both the S-LPS and R-LPS fractions of the wild-type strain were comparably stained with AgNO_3_, suggesting a similar abundance of these fractions. The staining intensity also reflects the susceptibility of C58Wt OPS to periodate oxidation, which occurs during the silver-staining process. The R-LPS fraction displayed two distinct bands on the polyacrylamide gel, indicating heterogeneity in the core oligosaccharide or variations in the lipid A structure, such as the presence or absence of specific fatty acids (e.g., 14:0-(3OH)). In contrast, the SDS-PAGE profile of the LPS from the C58Δfab mutant (Figure 3, lanes 6–8) differed significantly from that of the wild-type strain. The SR band was absent, and the R-LPS fraction exhibited faster-migrating subfractions that were more widely separated, indicating increased heterogeneity. Additionally, the S-LPS fraction of the mutant appeared less strongly stained and shifted to a higher molecular-weight region on the gel. Similar changes in LPS electrophoretic profiles have been observed and documented in rhizobial mutants of the *acpXL*–*lpxXL* gene cluster, highlighting the influence of these genes on LPS structure and properties [16,21,22,23].

### 2.4. Sensitivity of A. fabrum Mutants to Stress Conditions

Other authors observed that all mutants deficient in VLCFA biosynthesis were unable to survive beyond a few days on TY agar plates, and they produced minimal polysaccharide capsules in this medium. However, they were able to survive in the TY broth supplemented with divalent cations (Ca^2+^ and Mg^2+^) [8,17]. This phenomenon has been documented in VLCFA mutants of *S. meliloti* [21] and *R. leguminosarum* bv. Viciae [22]. Consequently, the mutants obtained in this work were cultured in the TY medium supplemented with 3.5 mM CaCl_2_ and 2.5 mM MgSO_4_ × 7H_2_O. Divalent cations are known to stabilise the OM by interacting with LPS molecules [24]. These results indicate that mutations eliminating VLCFAs from lipid A destabilise the OM, which can be partially compensated by the addition of agents that electrostatically interact with LPS.

A series of stressors, tested at various concentrations, showed that some affected the viability of the mutant cells. The results of the stress assays, summarised in Table 2, are presented as a percentage of the optical density at 600 nm (OD_600_) relative to control cultures of exact strain (cultures without stress-inducing agents). The data were collected at the mid-logarithmic growth phase of bacterial cultures (24–28 h of incubation). Stress-inducing agent concentrations were selected to allow for the significant viability of the wild-type strain. The mutants were hypersensitive to 0.5 mM SDS, 1 mM EDTA-Na_2_, partly to 100 μg/mL sodium deoxycholate (DOC), acidic (5.7 and 6.3) and basic pH (8.0). The mutants were much more sensitive to polymyxin B (PolyB) than wild-type strains. However, the growth of the C58Wt strain, mutants, and complemented derivatives at 37 °C was similar to normal conditions (28 °C). Taken together, these results suggest that the integrity of the OM is compromised in *A. fabrum* C58 mutants lacking VLCFAs.

### 2.5. Swimming Motility of A. fabrum Strains

**Figure 4:**
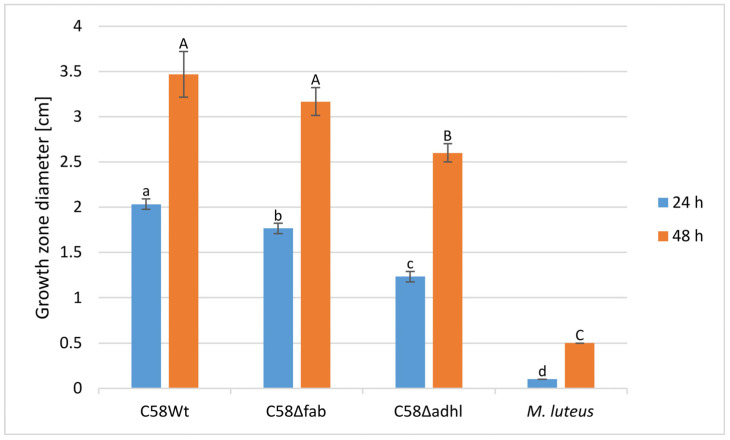
Swimming motility test of *A. fabrum* wild-type strain (C58Wt) and mutants (C58Δfab and C58Δadhl). *Micrococcus luteus*, a non-motile strain, was included as a negative reference. Growth zone diameters (in cm) were measured after 24 and 48 h of incubation. Data are presented as a mean ± SD from three independent experiments. Different letters indicate significant differences between groups: lowercase letters denote measurements taken after 24 h, while capital letters denote measurements taken after 48 h. Statistical significance was observed for both tested conditions (*p* < 0.001).

The swimming motility of the C58Δadhl mutant was substantially reduced compared to the wild-type strain, as evidenced by the smaller growth zone diameter observed in semi-solid agar plate tests, and this difference was significant after 24 and 48 h of incubation (Figure 4). In contrast, the swimming motility of the C58Δfab mutant was significantly affected relative to the wild-type strain only after 24 h of incubation. After 48 h, the growth zone diameter of the C58Δfab mutant was only slightly smaller than that of C58Wt. Moreover, C58Δfab demonstrated significantly greater motility than the C58Δadhl mutant strain, and this effect was observed at both 24 and 48 h of incubation.

### 2.6. Quantitative Analysis of A. fabrum Biofilm Formation on an Abiotic Surface and FTIR Spectroscopy Analysis of Biofilm Formed

**Figure 5:**
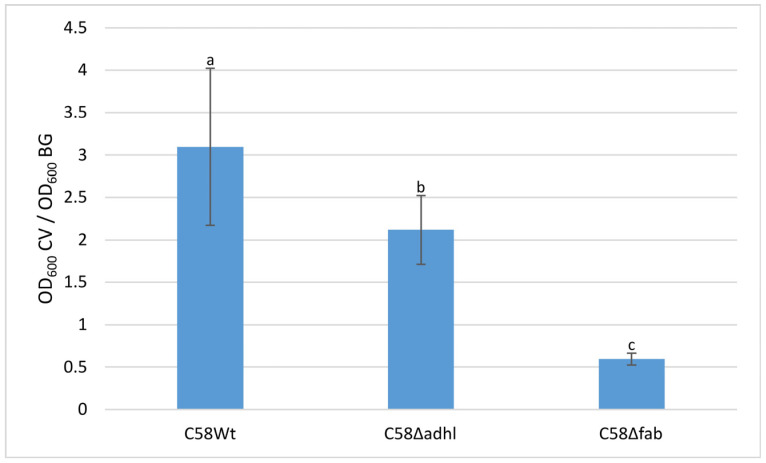
Quantitative analysis of biofilms produced by A. fabrum C58Wt and mutants: C58Δfab and C58Δadhl. Biofilm formation was assessed by staining with crystal violet and expressed as the ratio of the amount of crystal violet solubilised by ethanol to bacterial growth (OD600 CV/OD600 BG). Data were presented as a mean ± SD from three independent experiments. Different letters indicate significant differences between groups (*p* < 0.001).

To investigate the ability of *A. fabrum* C58Wt and its mutants VLCFA-deficient mutants to form biofilms on abiotic surfaces, bacteria were grown in 79CA medium in 24-well plates. The results showed that the biofilm formation of C58Wt was visibly higher than that of the mutants (Figure 5). The amount of biofilm formed by C58Δfab was significantly lower than that formed by C58Δadhl and C58Wt strains. Both mutants have impaired lipid A biosynthesis pathways that have an effect on the outer membrane properties and can influence the ability to adhere to an abiotic surface. In the case of the C58Δfab mutant, it seems that more bacterial cells remained in the planktonic form, which resulted in a high OD600 value for bacterial growth and significantly reduced the total measurement of biofilm amount.

**Figure 6:**
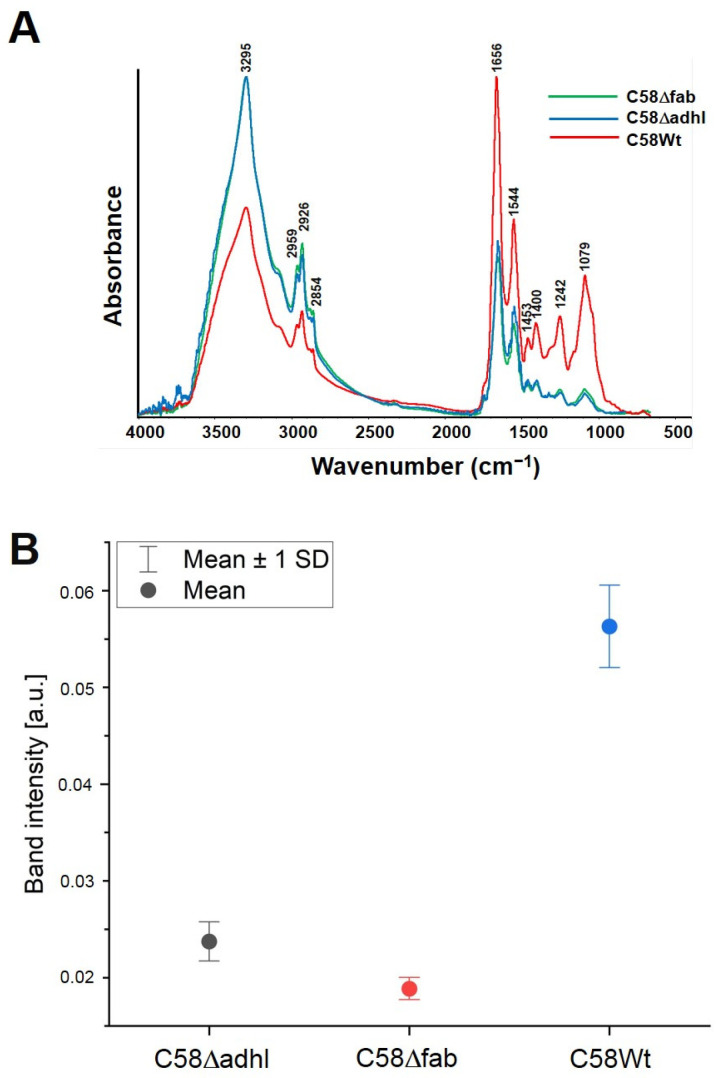
The vector normalised FTIR average spectra of each strain C58Δadhl, C58Δfab and C58Wt (**A**) and amide I (1650 cm^−1^) band intensity calculated from 4 random spectra from each sample with presented standard deviation (**B**). In the panel (**B**) mutant C58Δadhl was marked in gray, C58Δfab was marked in red wild strain C58Wt was marked in blue.

The averaged FTIR spectra of each sample indicating the corresponding wavenumbers for main peaks are presented in Figure 6A. There, our FTIR spectral band assignments to the typical vibrations in bacterial FTIR spectra were performed on the basis of other well-known studies [25,26,27]. The most significant changes are observed in the carbohydrate/phospholipid regions at about 1079 cm^−1^, and the highest intensity of the Amide I band (at 1656 cm^−1^) is observed in strain C58Wt. The mutants C58Δadhl and C58Δfab have very similar Amide I intensities (Figure 6B).

**Figure 7:**
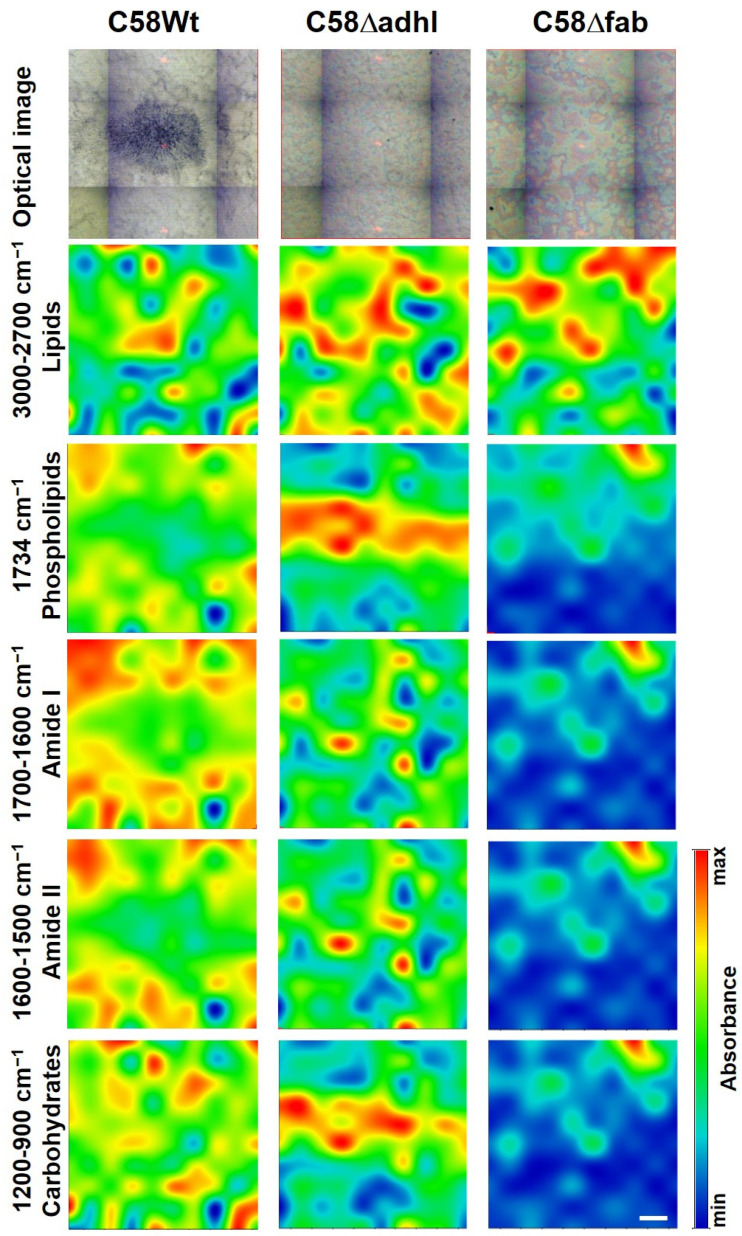
The FTIR chemical maps of compound distributions in biofilms formed by C58Wt, C58Δadhl, and C58Δfab *A. fabrum* strains, respectively. The white bar corresponds to 100 μm.

Spectroscopic mapping (FTIR analysis) of the distribution of proteins, lipids, and carbohydrates in the biofilm samples showed significant differences between both mutants and the C58Wt strain (Figure 7). Below are the optical images of biofilms formed by the tested strains on aluminium-coated glass slides. The images representing chemical maps of the surface distribution of selected components are displayed. The representative single FTIR spectra of biofilm formed by all the tested strains are presented in Supplementary Figure S4. The wavelength value (wavenumber) in the range of 3000–2700 cm^−1^ represents the stretching vibrations of CH_2_ and CH_3_ groups belonging to aliphatic chains of lipids, whereas those with a maximum at 1734 cm^−1^ are characteristic of stretching vibrations of carbonyl (C=O) groups in phospholipids [28,29]. Compared to the C58Wt, the biofilm produced by C58Δadhl showed more intense bands from lipids, whereas the biofilm produced by C58Δfab showed less intense bands, especially from phospholipids. The analysis of proteins present in the biofilms was based on the bands derived from two regions: Amide I (1700–1600 cm^−1^) and Amide II (1600–1500 cm^−1^), which are susceptible to changes in the secondary structures of proteins. The bands at 1700–1600 cm^−1^ derived mainly (in 80%) from stretching vibrations of C=O groups and in 20% from stretching vibrations of C-N linkages of Amide I, as well as from bending vibrations of water (H-O-H). In turn, the values of 1600–1500 cm^−1^ region usually derive 60% from bending vibrations of N–H groups, 30% from stretching vibrations of C–N linkages, and 10% from stretching vibrations of C–C linkages of Amide II [25,27]. The analysis of the obtained maps revealed that the biofilm formed by C58Wt was rich in proteins, indicating the presence of a high amount of bacterial cells, whereas the biofilm formed by the mutants (especially that with the mutation in the *fabF2XL* gene) revealed a very low intense signal from Amide I and II, suggesting a weak density of bacterial cells, and thus, indicating the low level of adhesion of the mutant cells to the abiotic surface. The analysis of the poly- and oligosaccharides present in the biofilm was based on the signals at the wavelength value of 1200–900 cm^−1^, where characteristic bands can be found deriving from stretching vibrations of C-C linkages in the carbohydrates skeleton (at 1080–1070 cm^−1^), skeletal vibration connected to anomeric structure of glucose (at 1050–1000 cm^−1^) and bands characteristic for α-glucans (at 930 cm^−1^) can be found [25,27]. The maps indicated that the biofilm formed by C58Wt was rich in carbohydrates, whereas the biofilm formed by mutant C58Δfab contained very low amounts of this type of component. The biofilm formed by C58Δadhl seems to have intermediate properties between the two discussed above.

### 2.7. Infection of Tomato Seedlings by A. fabrum Strains

**Figure 8:**
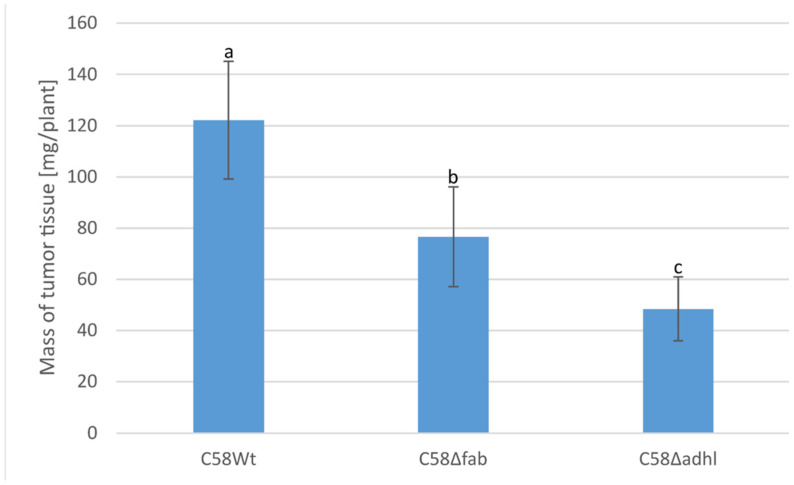
Average mass/plant of tumour tissue formed at young tomato seedlings (mean from 20 plants ± SD). Tumours were induced by C58Wt, C58Δfab and C58Δadhl strains, respectively. Different letters indicate significant differences between groups (*p* < 0.001).

The stems of tomato seedlings were infected by scarification with the C58Wt strain. Tumour tissue appeared two weeks after the day of infection and matured within the next two weeks; in the case of both mutants (C58Δadhl and C58Δfab), the tumours developed more slowly and became visible three weeks after infection. Representative plants with fully developed tumours were photographed (Supplementary Figure S5), and the tumour tissues were collected into separate Eppendorf tubes and weighted. The average mass of the tumour ± SD values are presented in Figure 8. It can be clearly seen that the tumour tissues from plants infected with C58Δfab and C58Δadhl were significantly smaller than those from the C58Wt strain. Moreover, the medium mass of tumour tissue was about 60% smaller in the case of plants infected with a mutant with an inactivated *adhlA2XL* gene compared to the plants infected with the C58Wt strain.

## 3. Discussion

All available data indicate that wild-type intracellular pathogens (*Legionella* and *Brucella*) and symbionts (rhizobia, except for *Azorhizobium caulinodans*) in the free-living state synthesise and incorporate VLCFAs into their lipids A. There is a generally accepted opinion that VLCFAs are components of lipid A/LPS also in the symbiotic stage of life. The investigations on the role of VLCFA in membrane maintenance, integrity, and properties were based on phenotypic analysis of mutants impaired in the synthesis or incorporation of VLCFAs into lipid A. To date, all available data related to the role of VLCFAs have been derived from mutational analysis of fast-growing rhizobia [8,30,31,32]. Mutants deprived of VLCFAs furnished their outer leaflet of OM with penta- and tetraacylated LPS molecules. In the case of the active *pagL* gene [33], even three-acylated lipid A molecules have been identified in OM. Generally, mutants with truncated lipid A (lack of VLCFA) had weakened outer membranes in relation to the wild-type strain and exhibited increased sensitivity to pH, detergents, peptide antibiotics, osmotic pressure, and desiccation [17,21,33,34]. Such mutants were impaired in symbiosis as well. It is interesting to notice that among many *S. meliloti* VLCFA mutants, those mutated in the *lpxXL* gene (coding for a specific acyltransferase) poorly tolerated harsh environmental conditions [17]. Their physiological properties were correlated with an inability to replace VLCFAs with any other fatty acids. Thus, *lpxXL* mutants synthesised lipid A with four or three (in the case of PagL action) acyl residues. Other mutants in the VLCFA gene cluster can produce pentaacyl lipid A, which reinforces OMs. Moreover, VLCFA gene cluster mutants required an elevated concentration of divalent cations in the growth medium [17,21]. In the case of insufficient concentrations of calcium and magnesium salts in the medium, *S. meliloti* VLCFA mutants required at least about 0.6% NaCl for growth [16]. A significant role in symbiosis and lipid A biosynthesis is attributed to the BacA protein [32].

The integrity of the bacterial OM is a function of the structure of particular lipid A. Furthermore, the OM integrity has a significant impact on the physiological properties of rhizobia and their ability to establish a close symbiotic interaction with legume plants. Although *A. fabrum* is most often classified together with other rhizobia in the same group of bacteria, they interact with plants typically as pathogenic bacteria. Agrobacteria are very similar to *S. meliloti* in the context of the OM structure, but they differ significantly in their relationship with plants (pathogen vs. symbiont). It can be hypothesised that the difference in the lipid A acylation pattern, which is further reflected in the OM structure, and its integrity constitute one of the most important factors influencing the interaction of pathogenic or symbiotic bacteria with the plant tissue. The structures of lipids A from *Agrobacterium* and *Sinorhizobium* are well known [10,16,21,32,35]. Moreover, *S. meliloti* mutants in all genes from VLCFA *loci* were obtained and characterised with respect to their physiological and symbiotic properties [16,17].

Although the *acpXL-msbB* region in *A. tumefaciens (A. fabrum)* has a very similar gene content and organisation as in *S. meliloti*, it was very interesting to compare *S. meliloti* and *A. fabrum* mutants in the same genes with respect to their impact on VLCFA biosynthesis, especially paying attention to the transcriptional organisation of the *acpXL-msbB (lpxXL)* region. The transcriptional organisation of the *acpXL*–*lpxXL* gene cluster in *A. fabrum* C58 differs from that previously reported in related bacteria. Our results demonstrate that *acpXL*–*lpxXL* gene cluster in *A. fabrum* C58 is organised into two transcriptional units: *acpXL*–*fabZXL* and *fabF2XL*–*fabF1XL*–*adhlA2XL*–*lpxXL*. In the homologous region of *S. meliloti* 1021, two transcriptional units were identified: one operon comprising *acpXL*–*fabZXL*–*fabF2XL*–*fabF1XL* and another one containing *adhlA2XL*–*lpxXL* genes [17]. In the corresponding gene cluster of *R. leguminosarum* bv. Viciae 3841, the *acpXL* gene is transcribed separately, three genes—*fabZ*, *fabF2*, and *fabF1*—are co-transcribed, while the gene coding for a putative dehydrogenase is transcribed separately from the former cluster [22]. These variations are likely to reflect species-specific regulatory adaptations to optimise VLCFA synthesis in distinct environmental conditions and alignment with symbiotic or pathogenic lifestyles.

This study was also focused on the role of two genes (*fabF2XL* and *adhlA2XL*) directly involved in the VLCFA biosynthetic pathway. We have proved that the activity of enzymes encoded by these genes is absolutely necessary for the proper synthesis thereof. The presence of VLCFAs in the structures of lipid A reinforcing the *A. fabrum* OM enables these bacteria to compete with other microorganisms in the natural environment. The inactivation of each of these genes in *A. fabrum* C58 induced greater susceptibility to almost all the stressors examined in the present study. Even the substitution of the missing VLCFA with typical cellular fatty acids (e.g., 18:0 or 18:1) was insufficient to compensate for this deficiency. The activity of LpxXL acyltransferase in both mutants results in the formation of lipid A molecules with acyloxyacyl moieties containing secondary fatty acids commonly found in *A. fabrum* cells (e.g., 16:0, 18:0 or 18:1), while the activity of LpxXL protein in the wild strain is demonstrated by the presence of VLCFA within lipid A. Moreover, the activity of PagL (NCBI database: *A. fabrum* strain C58, gene symbol ATU_RS09045; old locus: Atu1849) in *A. fabrum* is evidenced by the presence of tetraacylated lipid A (with fatty acid removed from C-3 position). Thus, the *A. fabrum* C58 mutants deprived of VLCFAs in their lipid A were considerably more sensitive to the stressing agents (e.g., EDTA, osmotic pressure, pH, SDS, elevated temperature, and polymyxin B). Their growth was delayed, and virulence was weakened (measured as the mass of tumours formed on tomato stems) compared to the wild-type strains (C58Wt). However, the salt tolerance in the presence of 2% NaCl and the high temperature of growth were at the same level as in the case of the C58Wt. To our surprise, the sensitivity of the C58Δadhl mutant to DOC was higher than that of the wild strain. This issue needs further investigation.

The interaction of the C58Δadhl and C58Δfab mutants with plastic (abiotic) surfaces was altered compared with wild-type cells. The weaker biological adsorption to plastic can affect the structure of biofilms [22]. The differences in biofilm formation were especially observed in the FTIR images. The low capacity in biofilm formation can be reflected in the low infectivity of the *A. fabrum* mutants.

Alterations in LPS structures were observed in the case of the C58Δfab mutant (SDS-PAGE). These alterations can cause OM modification and finally manifest themselves as motility-related defects (see: [22] and references therein). Further studies are needed to determine the changes in the OM that are a consequence of the lack of VLCFAs in *Agrobacterium* LPS.

## 4. Materials and Methods

### 4.1. Bacterial Strains and Culture Conditions

The bacterial strains used in this work are listed in Supplementary Table S1. *E. coli* strains were grown in lysogeny broth (LB) medium at 37 °C [36]. *A. fabrum* strains were cultivated at 28 °C in 79CA and TY complex media or M1 minimal medium, both supplemented with 1% (*w*/*v*) mannitol as the carbon source [37,38,39,40]. The composition of these media are listed in Supplementary Table S6. Mutant strains generated in this study were cultivated in 79CA medium supplemented with 3.5 mM CaCl_2_ and 2.5 mM MgSO_4_ × 7H_2_O (both from POCh, Gliwice, Poland) [21], referred to as 79CA/MC medium. Antibiotics were used at the following final concentrations: 100 μg/mL ampicillin, 40 μg/mL kanamycin, and 5 μg/mL (for *E. coli*) or 10 μg/mL (for *Agrobacterium*) gentamicin (each antibiotic was from MP Biochemicals, LLC, Solon, OH, USA).

### 4.2. Bioinformatic Analyses

Putative operons were identified using the Operon-mapper tool [40]. Promoter predictions within the *A. fabrum* C58 *acpXL*–*lpxXL* region (GenBank Accession: AE007869.2) were made using Neural Network Promoter Prediction [41] and PromoterHunter [41] tools. Rho-independent terminators were identified using the FindTerm algorithm [42]. General DNA sequence analyses and primer design were performed using SeqBuilder Pro v14.1.0 (DNASTAR, Inc., Madison, WI, USA). Significant differences among experimental variants were evaluated using one-way ANOVA followed by Tukey’s post hoc test conducted with R v4.2.3.

### 4.3. Total RNA Isolation and cDNA Synthesis

High-quality, DNA-free total RNA was isolated from *A. fabrum* C58 cells using a previously optimised protocol developed for *Rhizobium leguminosarum* bv. *trifolii* TA1 [18,19]. Briefly, *A. fabrum* C58 cells were grown overnight in 79CA medium at 28 °C with shaking, then diluted to an OD_600_ of 0.05 in fresh 79CA medium and incubated until reaching an OD_600_ of 0.6. Cells were harvested by centrifugation at 4 °C and immediately subjected to RNA extraction using the GeneMATRIX Universal RNA Purification Kit (EURx Sp. z o.o., Gdańsk, Poland), following the manufacturer’s protocol. Contaminating genomic DNA was removed using the TURBO DNA-free Kit (Thermo Fisher Scientific, Waltham, MA, USA) with rigorous DNase treatment. A total of three rounds of DNase treatment (2 U per round, for 30 min at 37 °C, in total 6 U and 90 min) were performed. The quantity and quality of RNA were assessed spectrophotometrically (Synergy H1 reader, Agilent Technologies, Inc., Santa Clara, CA, USA), fluorometrically (Qubit 2.0 Fluorometer with Qubit RNA High Sensitivity (HS) Assay Kit, Thermo Fisher Scientific, Waltham, MA, USA), and via microcapillary electrophoresis (2100 Bioanalyzer Instrument with RNA 6000 Nano Kit, Agilent Technologies, Inc., Santa Clara, CA, USA). The isolated DNA-free total RNA (0.5 µg) was used for reverse transcription with random primers, following the manufacturer’s recommendations (SuperScript IV VILO Master Mix with ezDNase Enzyme, Thermo Fisher Scientific, Waltham, MA, USA). The resulting cDNA was used as a template in a series of PCR reactions (PCR Mix Plus, A&A Biotechnology, Gdańsk, Poland) with primers specific to pairs of adjacent genes in the *A. fabrum* C58 *acpXL*–*lpxXL* region (Supplementary Table S2).

### 4.4. DNA Techniques

Genomic and plasmid DNA isolations were performed using the Bacterial and Yeast Genomic DNA Purification Kit (EURx Sp. z o.o., Gdańsk, Poland) and the Plasmid Miniprep DNA Purification Kit (EURx Sp. z o.o., Gdańsk, Poland), respectively, following the manufacturer’s protocols. Molecular cloning and transformation were carried out according to standard protocols [43]. FastDigest restriction endonucleases were obtained from Thermo Fisher Scientific (Waltham, MA, USA). PCRs were conducted using high-fidelity Platinum SuperFi II DNA Polymerase (Thermo Fisher Scientific, Waltham, MA, USA) or PCR Mix Plus (A&A Biotechnology, Gdańsk, Poland) according to the manufacturer’s instructions. The plasmids constructed and used in this study are listed in Supplementary Table S1. Primers used in this work were synthesised by Genomed S.A. (Warsaw, Poland) and are detailed in Supplementary Table S2. Sanger DNA sequencing of plasmid constructs was also performed by Genomed S.A. (Warsaw, Poland). Plasmids were introduced into *A. fabrum* C58 cells via electrotransformation, as described by Cooley et al. [44], with slight modifications. The final DNA concentration was adjusted to 1 ng/μL in the suspension of electrocompetent cells. After plasmid addition, the mixture was incubated on ice for 30 min, followed by electroporation using the ECM 630 electroporation system (BTX, Holliston, MA, USA) with 2 mm gap size cuvettes (Cell Projects Ltd., Harrietsham, UK). The electroporation parameters were set to 2.5 kV (corresponding to field strength of 12.5 kV/cm), 25 μF capacitance, and 200 Ω resistance. Immediately after the pulse, 1 mL of 79CA medium was added to the suspension, which was then incubated with shaking at 28 °C for 2–3 h before plating on selective media. Bacteria were grown on selective plates at 28 °C until single colonies of the desired size were obtained (3–7 days). Colonies were passed and subjected to further analysis.

### 4.5. β-Galactosidase Activity Measurements of Transcriptional Fusions

Plasmids carrying *lacZ* transcriptional fusions were constructed by cloning PCR-amplified fragments containing predicted promoter regions into the respective restriction sites of the pMPK vector [45] (Supplementary Table S1). The resulting plasmids were introduced into *A. fabrum* C58 cells via electroporation.

The level of *lacZ* expression was measured in Miller units by assaying β-galactosidase activity using ONPG (2-nitrophenyl-β-D-galactopyranoside, MP Biomedicals, LLC, Irvine, CA, USA) as a substrate, following the method described by Miller [46] with slight modifications. Strains harbouring *lacZ* transcriptional fusions in pMPK were grown overnight in 79CA medium supplemented with kanamycin. The cells were washed twice with sterile water, resuspended in fresh 79CA and M1 media, and grown to the mid-log phase. Cultures were cooled on ice for 20 min. A 200 μL aliquot of the suspension was transferred to a 96-well plate, and OD_600_ was measured (Synergy H1 reader, Agilent Technologies, Inc., Santa Clara, CA, USA). An appropriate volume of culture (0.1–0.5 mL) was diluted to 1 mL with cold Z buffer (60 mM Na_2_HPO_4_, 40 mM NaH_2_PO_4_, 10 mM KCl, 1 mM MgSO_4_, 50 mM 2-mercaptoethanol, pH 7.0) (all salts form POCh, Gliwice, Poland; 2-mercaptoethanol form Sigma-Aldrich, Saint Louis, MO, USA). To the diluted culture, 100 µL of chloroform (POCh, Gliwice, Poland) and 50 µL of 0.1% SDS (Sigma-Aldrich, Saint Louis, MO, USA) were added, followed by vigorous mixing for 10 s and incubation at 28 °C for 5 min. Subsequently, 200 µL of ONPG solution in Z buffer (4 mg/mL) was added to the samples, mixed, and incubated at 28 °C until a yellow colour developed. The reaction was terminated by the addition of 0.5 mL of 1 M Na_2_CO_3_ (POCh, Gliwice, Poland). Samples were centrifuged (5 min, 14,000× *g*), and 200 μL of the supernatant was collected for *OD*_420_ measurement. β-Galactosidase activity (in Miller units) was calculated using the equation:

$$U=\;\frac{1000\;\times \;{OD}_{420}}{{OD}_{600}\;\times reaction\;time\;\left(min\right)\times culture\;volume\;used\;in\;the\;reaction\;(mL)}$$

### 4.6. Construction of the fabF2XL and adhlA2XL Insertional Mutants and Their Complemented Derivatives

*A. fabrum* C58 insertional mutants for *fabF2XL* and *adhlA2XL* were generated using derivatives of pDOP [47] and pGEM-T Easy (Promega Corporation, Madison, WI, USA) plasmids, respectively (Supplementary Figures S1 and S2). A 777 bp fragment of the *fabF2XL* gene (spanning bp 324–1100, excluding bp 1–323 from the 5′ end of the gene and bp 1101–1197 from the 3′ end) and a 931 bp fragment of the *adhlA2XL* gene (spanning bp 1–803, excluding bp 804–1029 from the 3′ end) were amplified by PCR using *A. fabrum* C58 genomic DNA as a template. The *fabF2XL* fragment was cloned into the HindIII–BamHI sites of pDOP, resulting in pDfabF2XL. The *adhlA2XL* fragment was TA-cloned into pGEM-T Easy, resulting in pGTadhlA2XL. The correctness of the cloning was verified by colony PCR and restriction analysis. A gentamicin resistance cassette was amplified by PCR from the pBBR1-MCS5 plasmid [48] using primers equipped with SphI or HindIII sites. The cassette was then inserted into the SphI site of pDfabF2XL located within the *fabF2XL* fragment (resulting in pDfabF2XLGm) or into the HindIII site of pGTadhlA2XL, located within the *adhlA2XL* fragment (resulting in pGTadhlA2XLGm). The correctness of the constructs was confirmed by colony PCR, restriction analysis, and DNA sequencing. The plasmids were introduced into *A. fabrum* C58 recipient cells by electrotransformation and selected on solid 79CA/MC medium supplemented with gentamicin. Colonies were re-passaged on solid 79CA/MC medium with gentamicin and verified using PCR to confirm the disruption of the *fabF2XL* or *adhlA2XL* genes (Supplementary Figures S1 and S2).

For the construction of plasmids used in complementation analyses, a 1513 bp PCR fragment containing the *fabF2XL* gene and a 1445 bp PCR fragment containing the *adhlA2XL* gene were cloned into the XbaI site of the pBBR1-MCS2 vector under the P*lac* promoter [48], resulting in pBKfabF2XL and pBKadhlA2XL, respectively. The correctness of these constructs was confirmed by sequencing. The plasmids were then introduced into the respective mutant strains via electrotransformation, resulting in strains C58Δfab-C and C58Δadhl-C.

Bacterial strains, plasmids, and primers used or constructed during the mutagenesis procedure are listed in Supplementary Tables S1 and S2.

### 4.7. Lipopolysaccharide Fatty Acids Analysis

Bacteria (mutants and the wild strain) were grown in 79CA medium at 28 °C for 20 h. The 5 mL of the cultures were centrifuged, and the bacterial mass was delipidated three times by washing with 3 mL of chloroform/methanol (POCh, Gliwice, Poland) mixture (1:2, *v*/*v*) and centrifugation (4000× *g*, 15 min room temp.). Fatty acids from the delipidated bacterial mass were released through 18 h methanolysis using 1 mL of 2 M HCl/methanol at 85 °C. The reagents were removed in a nitrogen stream. Fatty acids methyl esters were recovered by extraction with 2 mL of chloroform/water (1:1, *v*/*v*) mixture and dried with anhydrous sodium sulphate (Sigma-Aldrich, Saint Louis, MO, USA). Free -OH groups of hydroxylated fatty acids were derivatised using 25 µL of trimethylsililating agent (Sylon HTP kit, Merck, Darmstadt, Germany), 15 min at room temperature. The products were analysed using a gas chromatograph coupled with a mass spectrometer (GC-MS) (GC System 7890A connected to a mass selective detector inert XL EI/CI MSD 5975C, Agilent Technologies, USA). The GC-MS system was equipped with an HP-5MS column (30 m × 0.25 mm), and helium was a carrier gas with a flow rate of 1 mL min^−1^. The temperature program was as follows: 150 °C for 5 min, raised to 310 °C at 5 °C min^−1^, and the final temperature was kept for 20 min. Fatty acid derivatives were identified based on their retention times and mass spectra using Enhanced Data Analysis software (Agilent Technologies, USA).

### 4.8. Isolation of Lipids A

Bacteria (mutants and wild strain) were grown at 79CA medium in 500 mL flasks in 100 mL of liquid medium at 28 °C for 20 h with aeration by vigorous shaking (160 rpm, INFORS HT Orbitron, Bottmingen, Switzerland). The bacteria were harvested, and the cell pellets were washed twice with saline. Lipids A preparations were obtained using a modified method described by Que and co-workers [49]. Briefly, each bacterial cell pellet was resuspended in 32 mL of phosphate-buffered saline (PBS, Sigma-Aldrich, Saint Louis, MO, USA), pH 7.4, and to extract the free lipids, the cell suspension was converted to a single-phase Bligh-Dyer mixture [50] by the addition of 40 mL of chloroform and 80 mL of methanol (both from POCh, Gliwice, Poland). After incubation for 1 h at room temperature with continuous stirring, the bacterial suspension was centrifuged at 5000× *g* for 15 min. The sediment was recovered and washed once with 38 mL of a fresh, single-phase Bligh-Dyer mixture consisting of chloroform/methanol/water (1:2:0.8 *v*/*v*/*v*). The insoluble material was again recovered by centrifugation, and the supernatant was discarded. The washed bacterial pellet containing LPS was then suspended in a 12-mL portion of 1% acetic acid (POCh, Gliwice, Poland). Dispersion was facilitated by a brief sonic irradiation in an ultrasonic bath. The ketosidic bond between the Kdo (2-keto-3-deoxyoconate) and the lipid A moiety was cleaved by heating the suspension to 100 °C in a boiling water bath for 1 h. After cooling, the suspension was converted to a two-phase Bligh-Dyer mixture by the addition of 40 mL of chloroform and 40 mL of methanol. The phases were mixed thoroughly and separated by centrifugation at 5000× *g* for 15 min at 25 °C. The lower (organic) phase containing the liberated lipid A was collected. A second extraction of the remaining upper phases was performed by adding 40 mL of a pre-equilibrated lower phase, which was obtained from a fresh two-phase Bligh-Dyer mixture consisting of chloroform/methanol/water (2:2:1.8 *v*/*v*/*v*) [49,50]. The mixture was again centrifuged to separate the phases, and the lower phases from the second extraction were pooled with the lower phases from the first extraction. After drying, the pooled lower phases were subjected to rotary evaporation in a round bottom flask at room temperature. The dried material was redissolved in 4 mL of chloroform/methanol (1:1 *v*/*v*), sealed, and stored at −20 °C.

### 4.9. MALDI-TOF Mass Spectrometry of Lipid A Preparations

MALDI-TOF mass spectrometry was performed with a SYNAPT G2-S*i* HDMS instrument (Waters Corporation, Milford, MA, USA) equipped with a 1 KHz Nd: YAG laser system (355 nm wavelength). Data acquisition was performed using MassLynx software version 4.1 (Waters Corporation, Wilmslow, UK). Mass spectra were assigned with a multi-point external calibration using red phosphorus (Sigma-Aldrich, Saint Louis, MO, USA). The lipid A samples were dissolved in chloroform/methanol (2:1, *v*/*v*) at a concentration of 20 µg/µL, and 2 mM of EDTA (Sigma-Aldrich, Saint Louis, MO, USA) was added. A sample (1 µL) was transferred into target plate wells covered with a thin matrix film. The matrix solution was prepared from 2′,4′,6′-trihydroxyacetophenone (THAP, Sigma-Aldrich, Saint Louis, MO, USA) (200 mg/mL in methanol) and mixed with nitrocellulose (NC, Whatman, Maidstone, UK) (15 mg/mL, suspended in 2-propanol/acetone (both from Sigma-Aldrich, Saint Louis, MO, USA), (1:1, *v*/*v*)) in a proportion of 4:1 (*v*/*v*), as described by Silipo and co-workers [51]. Spectra were recorded in positive and negative ion modes for 120 s (one scan per second).

### 4.10. Phenotypic Analysis of Bacteria

Mutants defective in VLCFA synthesis, strains with complementation of the mutation and the wild *A. fabrum* C58 strain were tested in terms of their physiological properties and resistance to stressing agents. Twenty-four-well plastic plates (Wuxi NEST Biotechnology, Wuxi, Jiangsu, China) were filled with 1 mL of TY medium [52] supplemented with tested components (each factor in triplicate) and inoculated with 50 μL of the bacterial strain (at OD_600_ = 0.1). Each strain was tested on a separate plate. Plates were incubated at 28 °C, and OD_600_ values were read using an automatic ELISA plate reader (ASYS UVM 340, Biogenet, Warszawa, Poland) after inoculation: (time 0), at 2 h, 4 h, 6 h, 8 h, 24 h, 28 h and 48 h. Between the measurements, the plates were placed in closed containers and aerated by gentle shaking using a table shaker (TR-250, INFORS HT, Bottmingen, Switzerland) at 110 rpm. Tested factors: sensitivity to salinity (with using 4%, 3%, 2%, 1%, 0.5%, 0.25% and 0% of NaCl in TY medium), sensitivity to sodium deoxycholate (Sigma-Aldrich, Saint Louis, MO, USA) (400, 300, 200, 100, 50, 25 and 0 μg/mL of DOC in TY medium), sensitivity to sodium dodecyl sulfate (Sigma-Aldrich, Saint Louis, MO, USA) (2, 1, 0.5, 0.25, 0.125 and 0 mM of SDS in TY medium); sensitivity to the chelating agent—ethylenediaminetetraacetic acid disodium salt (Sigma-Aldrich, Saint Louis, MO, USA) (2.00, 1.00, 0.5, 0.25, 0.125 and 0.00 mM of EDTA-Na_2_ in TY medium), sensitivity to pH (5.7; 6.3; 8.0, prepared based on TY medium containing phosphate buffer), presence of peptide antibiotics (25.0, 12.0, 6.0, 3.0, 1.0, 0.5 and 0.0 μg/mL of polymyxin B (Sigma-Aldrich, Saint Louis, MO, USA) in TY medium). Also, the sensitivity to elevated temperature (37 °C) was tested. Data were statistically analysed using one-way ANOVA with Tukey’s post hoc test, indicating differences at *p* < 0.005.

### 4.11. Swimming Motility Assay

Bacteria from an early logarithmic growth phase were spot-plated on the semi-solid 0.3% TY agar plates. *Micrococcus luteus* was used as a reference, a non-motile strain. The plates were incubated at 28 °C at 100% humidity and read after 24 and 48 h. Bacterial motility was determined by measuring the diameter of the circular zone covered by the bacteria [cm] [53].

### 4.12. Quantitative Analysis of A. fabrum Biofilm Formation on an Abiotic Surface

The quantification of the in vitro biofilm production was based on the modified method described previously [54,55]. Wells of 24-well flat bottom plates (Scienceplast, Gdańsk, Poland) were filled in triplicate with 2 mL of medium with a bacterial culture (OD_600_ = 0.1). The plates were incubated stationarily at 28 °C for 48 h, and OD_600_ was measured using an automatic ELISA plate reader (ASYS UVM 340, Biogenet, Warszawa, Poland). After removing the medium together with planktonic cells and rinsing the plates with PBS, the adherent cells were stained with 2 mL of 0.1% (*w*/*v*) crystal violet (CV, POCh, Gliwice, Poland) for 15 min, then CV was removed, and plates were rinsed twice with double-distilled water, and thoroughly dried. The CV dye bound to the adherent cells was solubilised with 80% (*v*/*v*) ethanol. After 10 min of incubation at room temperature, the OD_600_ of CV was measured to quantify the total biomass of biofilm formed in each well. Data were expressed as the ratio of the amount of crystal violet solubilised by ethanol (POCh, Gliwice, Poland) to bacterial growth (OD_600_ CV/OD_600_ BG). Data were presented as a mean ± SD from the three independent experiments.

### 4.13. FTIR Spectroscopy Analysis of A. fabrum Biofilm Formed on an Abiotic Surface

A set of 5.5 cm diameter plastic Petri dishes containing 6 mL of 79CA liquid medium were prepared and inoculated with 50 µL of the 20 h culture (OD_600_ = 0.1) of the wild strain and mutants in triplicates. A small part (2 cm × 1.5 cm) of a sterile aluminium-coated (thickness ~ 100 nm) Clear Borosilicate Float Glass Microscope Slide (Deposition Research Lab Inc., St. Charles, MO, USA) was immersed in each Petri dish, and the cultures were incubated at 28 °C for 48 h in stationary conditions. After incubation, the medium, together with planktonic cells, was removed, and the remaining biofilm adhering to the glass surface was rinsed with PBS and sterile water.

The semi-dry biofilms were measured using the FTIR (Fourier transform infrared) microspectroscopy technique, as was previously described [25]. FTIR spectra were collected in a transflection mode (i.e., by double pass through the sample) with the use of a Nicolet 6700 FTIR spectrometer (Thermo Scientific, Waltham, MA, USA) over the range of 4000–650 cm^−1^. The transflection mode is a combination of transmission and reflection. First, an IR beam passes through the sample, reflecting back from the metal surface (aluminium-coated slides), and comes back through the sample a second time [56]. Each spectrum represented an average of 120 scans taken. The spectra were registered at a resolution of 4 cm^−1^ with an optimal signal-to-noise ratio and an aperture of 100 μm. For area mapping, the X and Y step size was 150 µm × 150 µm. The objective magnification was set at 15×. Image assembly was performed using OMNIC 8.2.0.387 and CytoSpec Thermo Fisher Scientific, Madison, WI, USA) software.

### 4.14. Plant Infection Test

The stems of young tomato seedlings (6–8 weeks old) were inoculated with a suspension of *A. fabrum* C58 and mutants defective in VLCFA synthesis as was previously described [57]. A 20-gauge needle was pushed through the stem between the second and third leaf, making a wound c.a. 1 cm in length. A droplet (25 µL) of the bacterial suspension (OD_600_ = 0.1, in a mid-logarithmic growth phase) was extruded into the wound. Thirty plants were infected with each strain. The development of tumours was observed from 2 to 4 weeks after inoculation, and representative plants were photographed after 6 weeks. The complete, mature tumour tissue was then collected, and its fresh mass was estimated by its weighting. The mean ± SD of tumour tissue derived from 20 plants for each bacterial strain was calculated.

## 5. Conclusions

This study demonstrates the essential role of very long chain hydroxylated fatty acids (VLCFAs) in the lipid A structure of *Agrobacterium fabrum* and their impact on the overall bacterial physiology. The absence of VLCFAs, due to mutations in the *fabF2XL* or *adhlA2XL* genes, resulted in impaired bacterial growth, increased susceptibility to environmental stressors, defective biofilm formation, and reduced swimming motility. Notably, the mutants exhibited a significantly weakened ability to infect tomato seedlings, highlighting the importance of an intact OM structure for effective pathogenic interactions.

Our findings underscore the critical role of VLCFAs in maintaining OM integrity, which directly influences bacterial survival in soil environments and interactions with host. The observed structural modifications in lipid A and subsequent physiological defects in mutants provide compelling evidence that VLCFA incorporation is a key determinant of *A. fabrum* virulence. Moreover, the partial restoration of wild-type characteristics in complemented strains suggests that proper lipid A acylation is essential for bacterial resilience.

Overall, this work contributes to a deeper understanding of the molecular mechanisms governing OM stability in *A. fabrum* and reinforces the broader significance of lipid A modifications in Gram-negative bacterial adaptation. Future studies should explore the regulatory pathways governing VLCFA biosynthesis and their potential as targets for controlling *A. fabrum* infections in agricultural settings.

## Figures and Tables

**Table 1:** Fatty acids composition of lipid A preparations from *A. fabrum* C58 (C58Wt) and mutants in *fabXF2* and *adhl2AXL* genes (C58Δfab and C58Δadhl) and their complementants (C58Δfab-C and C58Δadhl-C). Fatty acid percentages are calculated based on GC-MS chromatograms.

	Strain	C58Wt	C58Δfab	C58Δfab-C	C58Δadhl	C58Δadhl-C
Fatty Acid						
16:0		5.8	10.5	5.9	6.7	4.3
14:0-(3OH)		23.9	33.0	8.8	44.4	21.4
18:2		1.4	0.1	10.0	0.1	6.7
18:1 ^1^		8.7	8.9	45.1	4.4	24.4
18:1 ^2^		15.3	12.9	11.2	15.2	11.3
18:0		6.3	6.7	7.1	0.7	4.0
16:0-(3OH)		20.4	16.7	7.1	21.8	16.1
18:1-(3OH)		2.6	8.8	Tr.	6.5	4.0
18:0-(3OH)		2.7	2.4	Tr.	0.1	2.9
26:0-(25-OH)		-	-	-	-	2.3
28:0-(27OH)		11.0	-	4.8	-	2.7
30:0-(29OH)		2.0	-	-	-	-

^1, 2^ Two isoforms of 18:1. Tr.—traces (below 0.1%).

**Table 2:** Sensitivity of *A. fabrum* derivatives to stress-inducing factors. Data are presented as a percentage of OD_600_ value normalised to the control culture (culture without stress-inducing factor) ± standard deviation (SD).

Strain	NaCl 2%	DOC 100 µg/mL	EDTA-Na_2_ 1 mM	SDS 0.5 mM	pH 5.7	pH 6.3	pH 8.0	PolyB 6 µg/mL	Elevated Temperature from 28 °C to 37 °C
C58Wt	162 ^A^ ± 3	97 ^A^ ± 2	117 ^Aa^ ± 4	101 ^Aa^ ± 3	70 ^a^ ± 2	70 ^a^ ± 2	80 ^Aa^ ± 9	89 ^Aa^ ± 1	87 ± 5
C58Δfab	132 ^B^± 10	70 ^B^ ± 4	91 ^B^ ± 2	18 ^B^ ± 0.5	69 ± 2	71 ± 1	66 ^B^ ± 2	60 ^B^ ± 5	82 ± 10
C58Δfab-C	96 ^C^ ± 8	110 ^C^ ± 5	96 ^B^ ± 4	37 ^C^ ± 3	71 ± 2	75 ± 2	81 ^A^ ± 2	70 ^B^ ± 8	91 ± 4
C58Δadhl	147 ± 14	124 ± 10	80 ^b^ ± 4	24 ^b^ ± 2	51 ^b^ ± 3	50 ^b^ ± 4	48 ^b^ ± 5	34 ^b^ ± 2	98 ± 8
C58Δadhl-C	138 ± 14	117 ± 15	85 ^b^ ± 5	85 ^a^ ± 9	58 ^b^ ± 3	56 ^b^ ± 1	61 ^c^ ± 0.5	46 ^c^ ± 5	81 ± 1

Abbreviations used: DOC—sodium deoxycholate; EDTA-Na_2_—ethylenediaminetetraacetic acid disodium salt; SDS—sodium dodecyl sulfate; PolyB—polymyxin B. Different letters in superscripts within the same column indicate significant differences at *p* < 0.005 (one-way ANOVA with Tukey’s post hoc test). Capital letters denote comparisons between the wild-type strain and Δ*fabF2* derivatives, while lowercase letters denote comparisons between the wild-type strain and Δ*adhlA2* derivatives).

## Data Availability

The raw data supporting the conclusions of this article will be made available by the authors upon request.

## Supplementary Materials

The following supporting information can be downloaded at https://www.mdpi.com/article/10.3390/molecules30051080/s1, Table S1: Bacterial strains and plasmids used in this work; Table S2: Primers used in this study; Table S3: In silico prediction of promoters in the *A. fabrum* C58 acpXL–lpxXL region; Table S4: In silico prediction of Rho-independent terminators in the *A. fabrum* C58 acpXL–lpxXL region; Figure S1: Schematic representation of the steps involved in the construction of the *A. fabrum* C58 *fabF2XL* insertional mutant (A) and the results of PCR confirming successful mutagenesis (B). The following primer pairs were used for DNA amplification: fabF2-O_Fw and M13pUCr for fragment 1, M13pUCf and fabF2XL-O_Fw for fragment 2, M13pUCf and GmFw for fragment 3, and M13pUCf and GmRv for fragment 4. The last two primer pairs were used to confirm the orientation of the gentamicin resistance cassette within f*abf2XL*; Figure S2: Schematic representation of the steps involved in the construction of the *A. fabrum* C58 f*abF2XL* insertional mutant (A) and the results of PCR confirming successful mutagenesis (B). The following primer pairs were used for DNA amplification: fabF2-O_Fw and M13pUCr for fragment 1, M13pUCf and fabF2XL-O_Fw for fragment 2, M13pUCf and GmFw for fragment 3, and M13pUCf and GmRv for fragment 4. The last two primer pairs were used to confirm the orientation of the gentamicin resistance cassette within *fabf2XL*; Table S5: Masses and proposed compositions of selected ions observed in MALDI-TOF MS of intact lipids A isolated from *A. fabrum* C58 and its mutants and complementants and examples of fragment ions; Figure S3: Proposed structures of the main species of lipid A identified in: A) *A. fabrum* C58Wt and B) mutant strains, deprived of VLCFAs and two examples of fragment ions with double bonds within sugar rings; Figure S4: Representative FTIR spectra of biofilm formed at the abiotic surface by *A. fabrum* C58Wt (red line) and mutants C58Δadhl (blue line) and C58Δfab (green line). Figure S5: Tomato seedlings infected with *A. fabrum* C58Wt and mutants C58Δfab and C58Δadhl. Plant stems were photographed 6 weeks after infection. A ruler placed next to the seedling allows us to estimate the size of changes on tomato stems. Table S6: Composition of the bacterial media used in this study.
