# Supplementary material for: The Absence of a Very Long Chain Fatty Acid (VLCFA) in Lipid A Impairs Agrobacterium fabrum Plant Infection and Biofilm Formation and Increases Susceptibility to Environmental Stressors
Source: Molecules. 2025 Feb 26;30(5):1080. doi: 10.3390/molecules30051080 (PMC11901934; doi:10.3390/molecules30051080)
Supplement: Supplementary file 1 [file molecules-30-01080-s001.zip › molecules-3479571-supplementary.pdf]

Supplementary Materials

The absence of a very long-chain fatty acid (VLCFA) in lipid A impairs *Agrobacterium fabrum* plant infection and biofilm formation, and increases susceptibility to environmental stressors

Iwona Komaniecka[1*], Kamil Żebracki[1], Andrzej Mazur[1], Katarzyna Suśniak[2], Anna Sroka-Bartnicka[3], Anita Swatek[1], Adam Choma[1*]

[1]Department of Genetics and Microbiology, Institute of Biological Sciences, Maria Curie-Sklodowska University, Lublin, Poland; iwona.komaniecka@mail.umcs.pl, kamil.zebracki@mail.umcs.pl, andrzej.mazur@mail.umcs.pl, anita.swatek@mail.umcs.pl

[2] Department of Pharmaceutical Microbiology, Medical University of Lublin, Chodźki 1, PL-20-093 Lublin, Poland. katarzyna.susniak@umlub.pl

[3] Independent Unit of Spectroscopy and Chemical Imaging, Medical University of Lublin, Chodźki 4a Street, 20-093 Lublin, Poland. anna.sroka-bartnicka@umlub.pl

Supplementary content:

Table S1. Bacterial strains and plasmids used in this work

Table S2. Primers used in this study

Table S3. *In silico* prediction of promoters in the *A. fabrum* C58 *acpXL–lpxXL* region

Table S4. *In silico* prediction of Rho-independent terminators in the *A. fabrum* C58 *acpXL–lpxXL* region

Figure S1. Schematic representation of the steps involved in the construction of the *A. fabrum* C58 *fabF2XL* insertional mutant (**A**) and the results of PCR confirming successful mutagenesis (**B**). The following primer pairs were used for DNA amplification: fabF2-O_Fw and M13pUCr for fragment 1, M13pUCf and fabF2XL-O_Fw for fragment 2, M13pUCf and GmFw for fragment 3, and M13pUCf and GmRv for fragment 4. The last two primer pairs were used to confirm the orientation of the gentamicin resistance cassette within *fabf2XL*

Figure S2. Schematic representation of the steps involved in the construction of the *A. fabrum* C58 *adhlA22XL* insertional mutant (**A**) and the results of PCR confirming successful mutagenesis (**B**). The following primer pairs were used for DNA amplification: adhlA2-O_Fw and M13pUCr for fragment 1, M13pUCf and adhlA2XL-O_Fw for fragment 2, M13pUCf and GmFw for fragment 3, and M13pUCf and GmRv for fragment 4. The last two primer pairs were used to confirm the orientation of the gentamicin resistance cassette within *adhlA2XL*.

Table S5. Masses and proposed compositions of selected ions observed in MALDI-TOF MS of intact lipids A isolated from *A. fabrum* C58 and its mutants and complementants with examples of fragment ions.

Figure S3. Proposed structures of the main species of lipid A identified in: **A)** *A. fabrum* C58 Wt and **B)** mutant strains deprived of VLCFAs and two examples of fragment ions with double bonds within sugar rings.

Figure S4. Representative FTIR spectra of biofilms formed at the abiotic surface by *A. fabrum* C58Wt and mutants C58Δfab and C58Δadhl strains.

Figure S5. Tomato seedlings infected with *A. fabrum* C58Wt and mutants C58Δfab and C58Δadhl. Plant stems were photographed 6 weeks after infection. A ruler placed next to the seedling allows estimating the size of changes on the tomato stems.

Table S6. Composition of the bacterial media used in this study.

Supplementary Table S1. Bacterial strains and plasmids used in this work

Strains or plasmids	Description	Source/Reference
Escherichia coli		
DH5α	F^- φ80*lacZ*ΔM15 Δ(*lacZYA–argF*) U169 *recA1 endA1 hsdR17* (r_k^-, m_k^+) *phoA supE44* λ^- *thi*-1 *gyrA96 relA1*, high-efficiency transformation strain	Thermo Fisher Scientific (Waltham, MA, USA)
Agrobacterium fabrum		
C58Wt	Wild-type pathogenic strain, carries pTiC58 and pAtC58	ATCC 33970 (Manassas, VA, USA)
C58Δfab	C58Wt Δ*fabF2XL*::pDfabF2XLGm, Gen^R	This study
C58Δfab-C	Complementary strain of C58Δfab obtained by transforming pBKfabF2XL into C58Δfab, Gen^R, Km^R	This study
C58Δadhl	C58Wt Δ*adhlA2XL*:: pGTadhlA2XLGm, Gen^R	This study
C58Δadhl-C	Complementary strain of C58Δadhl obtained by transforming pBKadhlA2XL into C58Δadhl, Gen^R, Km^R	This study
Plasmids used for promoter activity assay		
pMPK	IncP, *mob*, promoterless *lacZ*, Tc^R, Km^R	Żebracki et al., 2015
pPacpXL1	pMPK with a 171 bp KpnI–XbaI fragment comprising the last 22 bp of *hemN*, a *hemN–acpXL* intergenic region, and the initial 35 bp of *acpXL*	This study
pPacpXL2	pMPK with a 127 bp KpnI–XbaI fragment comprising 92 bp upstream of *acpXL* and the initial 35 bp of *acpXL*	This study
pPacpXL3	pMPK with a 75 bp KpnI–XbaI fragment comprising 40 bp upstream of *acpXL* and the initial 35 bp of *acpXL*	This study
pPfabZXL	pMPK with a 204 bp KpnI–XbaI fragment comprising 76 bp upstream of *fabZXL* and the initial 128 bp of *fabZXL*	This study
pPfabF2XL1	pMPK with a 271 bp KpnI–XbaI fragment comprising 207 bp upstream of *fabF2XL* and the initial 64 bp of *fabF2XL*	This study
pPfabF2XL2	pMPK with a 185 bp KpnI–XbaI fragment comprising 121 bp upstream of *fabF2XL* and the initial 64 bp of *fabF2XL*	This study
pPfabF2XL3	pMPK with a 152 bp KpnI–XbaI fragment comprising 88 bp upstream of *fabF2XL* and the initial 64 bp of *fabF2XL*	This study
pPadhlA2XL	pMPK with a 301 bp KpnI–XbaI fragment comprising the last 106 bp of *fabF1XL*, *fabF1XL–adhlA2XL* intergenic region, and the initial 60 bp of *adhlA2XL*	This study

Plasmids used for construction of insertional mutants and their complemented derivatives		
pGEM-T Easy	ColE1 *ori*, *lacZα* multi-cloning site, f1 *ori*, Ap^R, contains 3′-T overhangs, suicide vector in *Agrobacterium* cells	Promega Corporation (Madison, WI, USA)
pDOP	Derived from pBC SK(+), *oriT*, Cm^R, suicide vector in *Agrobacterium* cells	Cervantes-Rivera, et al., 2011
pBBR1-MCS2	pBBR1 *rep*, *mob*, *lacZα* multi-cloning site, Km^R, broad-host-range cloning vector	Kovach et al., 1995
pBBR1-MCS5	pBBR1 *rep*, *mob*, *lacZα* multi-cloning site, Gm^R, broad-host-range cloning vector	Kovach et al., 1995
pDfabF2XL	pDOP with a 777 bp HindIII–BamHI fragment comprising bp 324–1100 of *fabF2XL*	This study
pGTadhlA2XL	pGEM-T Easy with a 931 bp TA-cloned fragment comprising 128 bp upstream of *adhlA2XL* and the initial 803 bp of *adhlA2XL*	This study
pDfabF2XLGm	pDfabF2XL with a gentamicin resistance cassette from pBBR1-MCS5 cloned into the SphI site within the *fabF2XL* fragment	This study
pGTadhlA2XLGm	pGTadhlA2XL with a gentamicin resistance cassette from pBBR1-MCS5 cloned into the HindIII site within the *adhlA2XL* fragment	This study
pBKfabF2XL	pBBR1-MCS5 with a 1513 bp XbaI fragment comprising 207 bp upstream of *fabF2XL*, *fabF2XL*, *fabF2XL– fabF1XL* intergenic region, and the initial 97 bp of *fabF1XL*	This study
pBKadhlA2XL	pBBR1-MCS5 with a 1445 bp XbaI fragment comprising the last 106 bp of *fabF1XL*, *fabF1XL– adhlA2XL* intergenic region, *adhlA2XL*, and the initial 172 bp of *lpxXL*	This study

Supplementary Table S2. Primers used in this study

Primers	Sequence (5′–3′) [1]	Reference	Description
Primers used for RT-PCR analysis of the A. fabrum C58 acpXL–lpxXL region			
hemN-RT_Fw	GAAAGGCTGATGTGCGATTTCTCTATC	This study	To amplify the last 243 bp of *hemN*, *hemN–acpXL* intergenic region, and the initial 193 bp of *acpXL*
acpXL-RT_Rv	TCGTCGATCGTGTGGCTCTCC	This study	
acpXL-RT_Fw	CAAGGTTTCCACCGAAGAATACTT	This study	To amplify the last 79 bp of *acpXL*, *acpXL–fabZXL* intergenic region, and the initial 325 bp of *fabZXL*
fabZXL-RT_Rv	TGGTAACGGCAAAGCCCGAAC	This study	
fabZXL-RT_Fw	TGAAGGCGCAGTCGGTCGTT	This study	To amplify the last 416 bp of *fabZXL*, *fabZXL–fabF2XL* intergenic region, and the initial 64 bp of *fabF2XL*
fabF2XL-RT_Rv	TGCCAACGCCGTGACAGGT	This study	
fabF2XL-RT_Fw	CGGCGCGAGCGGTTATGAC	This study	To amplify the last 289 bp of *fabF2XL*, *fabF2XL–fabF1XL* intergenic region, and the initial 97 bp of *fabF1XL*
fabF1XL-RT_Rv	CCCAGTTGTCGGCAAGTCCCT	This study	
fabF1XL-RT_Fw	GGTGCCGAATGTGAAGCGGGATGC	This study	To amplify the last 106 bp of *fabF1XL*, *fabF1XL–adhlA2XL* intergenic region, and the initial 60 bp of *adhlA2XL*
adhlA2XL-RT_Rv	GGGTTCAGGCAGGTCCACTTTTT	This study	
adhlA2XL-RT_Fw	GCGCGGCATCGTTCATCC	This study	To amplify the last 118 bp of *adhlA2XL* and the initial 171 bp of *lpxXL*
lpxXL-RT_Rv	GCTTGATAGGGCCGAAAACCG	This study	
lpxXL-RT_Fw	CACCAGATTTGAGGCGATTGC	This study	To amplify the last 12 bp of *lpxXL*, *lpxXL–Atu1592* intergenic region, and the initial 314 bp of *Atu1592*
Atu1592-RT_Rv	AGCGTATCCTCGTCCTCGTC	This study	
Primers used for promoter activity assay			
PacpXL1_FwKpn	aaaggtaccCCGTCATTCGGTTGCTGTTTAGGC	This study	To amplify the upstream fragments of *acpXL* and insert into pMPK
PacpXL2_FwKpn	aaaggtaccCAAAGTTACGTCACGAAACCATTG	This study	
PacpXL3_FwKpn	aaaggtaccGCGTCAGAAAGAACAATTCATACG	This study	
PacpXL_RvXba	aatctagaTCGGCAACCTTGTCGAATGTAGC	This study	
PfabZXL_FwKpn	aaaggtaccGCATCGAGCGGGCATTTCTT	This study	To amplify the upstream fragment of *fabZXL* and insert into pMPK
PfabZXL_RvXba	aatctagaGGCATGCCGGGAAAGTGG	This study	
PfabF2XL1_FwKpn	aaaggtaccCGCTTCCGCCCGTTTCAA	This study	To amplify the upstream fragments of *fabF2XL* and insert into pMPK
PfabF2XL2_FwKpn	aaaggtaccGCCTTCGCCATTGCCAATCC	This study	

PfabF2XL3_FwKpn	aaaggtaccTATTCCGCAACGCATGAAGTGGTT	This study	
PfabF2XL_RvXba	aatctagaTGCCAACGCCGTGACAGGT	This study	
PadhlA2XL_FwKpn	aaaggtaccGGTGCCGAATGTGAAGCGGGATGC	This study	To amplify the upstream fragment of *adhlA2XL* and insert into pMPK
PadhlA2XL_RvXba	aatctagaGGGTTCAGGCAGGTCCACTTTTT	This study	
Primers used for construction of insertional mutants and complementing plasmids			
fabF2XL_FwHind	aaaaagcttCGGCGAGCGCGACATCAAT	This study	To construct the pDfabF2XLGm vector for insertional mutagenesis of *fabF2XL*
fabF2XL_RvBam	aaggatccCGCAGCCGCATCGAAGGTAGGAAC	This study	
GmFwSph	agatagcatgcAAACGGCATGATGAACCTGAATC	This study	
GmRvSph	agatagcatgcTCTCGGCTTGAACGAATTGTTAGG	This study	
adhlA2XL_Fw	CGGGCCAGCTCGTTTCGGTCTATG	This study	To construct the pGTadhlA2XLGm vector for insertional mutagenesis of *adhlA2XL*
adhlA2XL_Rv	CCCGAGGTGGAGCCGCAGGTGA	This study	
GmFwHind	aaaaagcttAAACGGCATGATGAACCTGAATC	This study	
GmRvHind	aaaaagcttTCTCGGCTTGAACGAATTGTTAGG	This study	
fabF2XL-C_FwXba	aatctagaCGCTTCCGCCCGTTTCAA	This study	To amplify the *fabF2XL* DNA fragment with its native promoter and insert into pBBR1-MCS5
fabF2XL-C_RvXba	aatctagaCCCAGTTGTCGGCAAGTCCCT	This study	
adhlA2XL-C_FwXba	aatctagaGGTGCCGAATGTGAAGCGGGATGC	This study	To amplify the *adhlA2XL* DNA fragment and insert into pBBR1-MCS5
adhlA2XL-C_RvXba	aatctagaCGGTTTTCGGCCCTATCAAGC	This study	
fabF2XL-O_Fw	GCATGTGGCGCTTCTTTCTGGA	This study	To verify successful insertional mutagenesis of *fabF2XL* and *adhlA2XL*
fabF2XL-O_Rv	CTTCGAGCGATTCCAACACCA	This study	
adhlA2XL-O_Fw	GGTGCCGAATGTGAAGCGGGATGC	This study	
adhlA2XL-O_Rv	CGAGATTGCCCATGCGTTCCTT	This study	
GmFw	AAACGGCATGATGAACCTGA	This study	
GmRv	TCTCGGCTTGAACGAATTGTTAGG	This study	
M13pUCf	CCCAGTCACGAAGTTGTAAAACG	Universal	To validate cloning and sequencing of

		primer	pBBR1-MCS2 derivatives and verify successful insertional mutagenesis
M13pUCr	AGCGGATAACAATTTCACACAGG	Universal primer	

[1] The underlines indicate restriction sites.

Supplementary Table S3. *In silico* prediction of promoters in the *A. fabrum* C58 *acpXL–lpxXL* region

Locus	DNA region [1]	Sequence of the hypothetical promoter [2]	N [3]	P [4]
acpXL	-125 – -94	TTGCTGTTTAGGCAACAGTTTTGGGGCAAATT	20	0.61
	-53 – -24	TTGCCTATGTGGTGCGTCAGAAAGAACAAT	18	0.81
	-36 – -8	CAGAAAGAACAATTCATACGAACGACAAT	17	0.53
fabZXL	-88 – -7	CTGACAATGCCCGCATCGAGCGGGCATTTCTT	20	0.11
fabF2XL	-178 – -146	TTTTCCGTCGGCCCGGTTTCGATGCATGAAAAT	21	0.74
	-111 – -85	TTGCCAATCCGCATCGCGCGCTTTATT	15	0.68
	-41 – -9	GTGACCGTTTCGGCGGGGCCGGCTGCAAAGGAT	21	0.18
adhlA2XL	-119 – -90	TTTCCAAAAGCACTGCGGAAACGCTCTCGT	18	0.17
	-58 – -27	GTTACAGAGTTTTGCTGGAATGATTTTTAGAT	20	0.11
	-33 – -1	TTTAGATAACGCCCTCGGGCGAAGGACTGAAAT	21	0.96

[1] Positions of predicted promoters are given relative to the ATG codon of the preceding gene.
[2] Hypothetical −35 and −10 elements are highlighted in green and blue, respectively.
[3] Spacing between the −35 and −10 sequences in base pairs
[4] Probability that a given sequence functions as a promoter, with a score 1.0 indicating the highest probability

Supplementary Table S4. *In silico* prediction of Rho-independent terminators in the *A. fabrum* C58 *acpXL*–*lpxXL* region

Intergenic locus	Region [1]	Sequence of the hypothetical terminator [2]	N [3]	Score [4]
acpXL– fabZXL	9–36	TGCCCGCATCGAGCGGGCATTTCTTTTT	28	-16.50
fabZXL– fabF2XL	8–34	CGCCCGTTTCAAAAGAGGGCGTTTTTT	27	-9.20
fabF1XL–adhlA2XL	67–116	CATTCCAGCCGCAAAACCGTTACAGAGTTTTGCTGGAATG ATTTTTAGAT	50	-14.40
lpxXL–Atu1592	46–81	ACCGCGAATTCGTGCATTTCGGTATTTTTTTATATT	36	-5.60

[1] Positions of predicted terminators are relative to the stop codon of the gene preceding the terminator, spanning from the start of the hairpin structure to the end of the T-rich region.
[2] Colored secondary structures: predicted hairpin stems are represented in blue and loops are in green.
[3] The total length of the terminator measured in base pairs from the start of the hairpin structure to the end of the T-rich region.
[4] The value of the scoring function, which incorporates the energy of the terminator. Lower scores indicate more stable terminators.

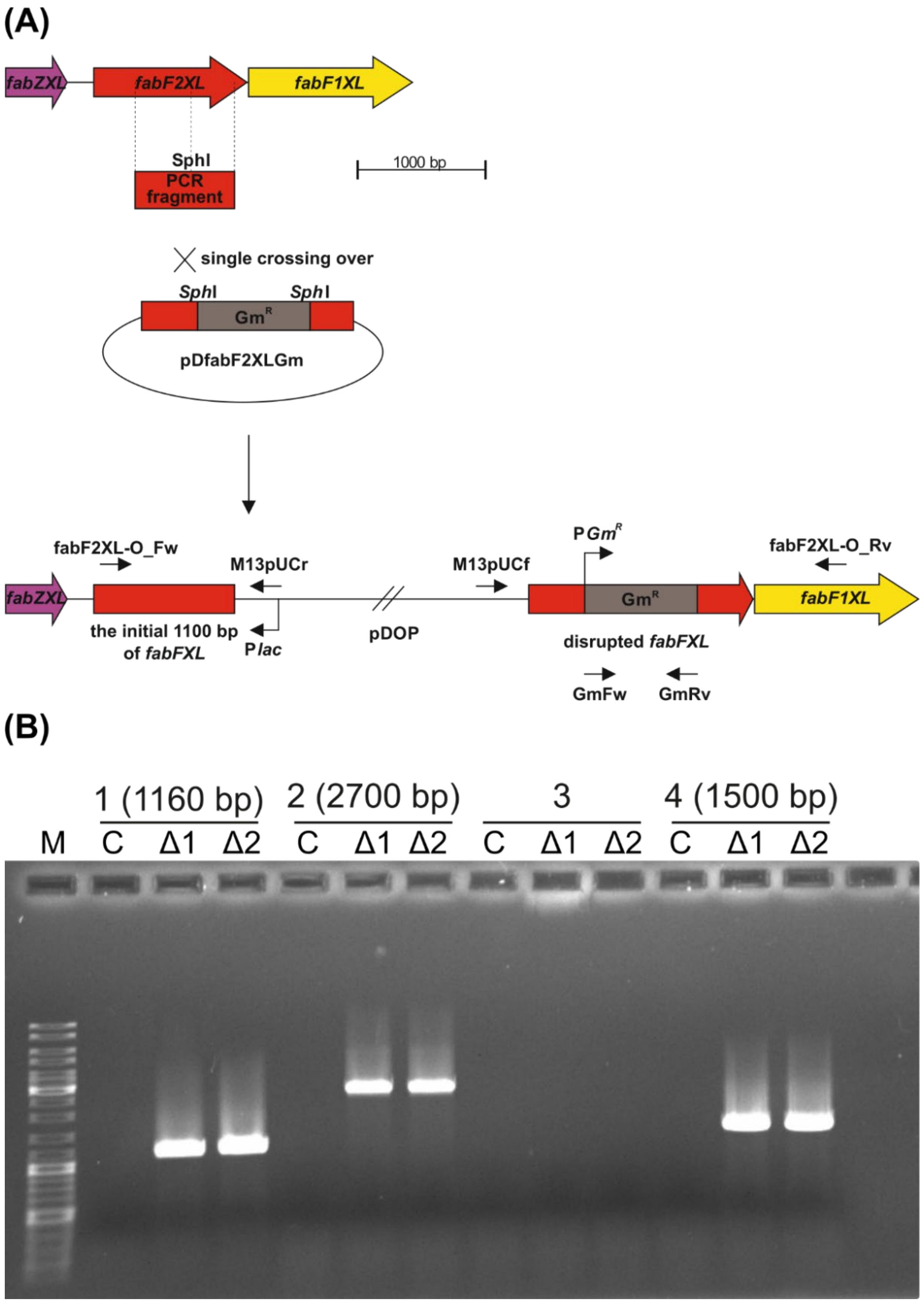

Supplementary Figure S1. Schematic representation of the steps involved in the construction of the *A. fabrum* C58 *fabF2XL* insertional mutant (**A**) and the results of PCR confirming successful mutagenesis (**B**). The following primer pairs were used for DNA amplification: fabF2-O_Fw and M13pUCr for fragment 1, M13pUCf and fabF2XL-O_Fw for fragment 2, M13pUCf and GmFw for fragment 3, and M13pUCf and GmRv for fragment 4. The last two primer pairs were used to confirm the orientation of the gentamicin resistance cassette within *fabf2XL*.

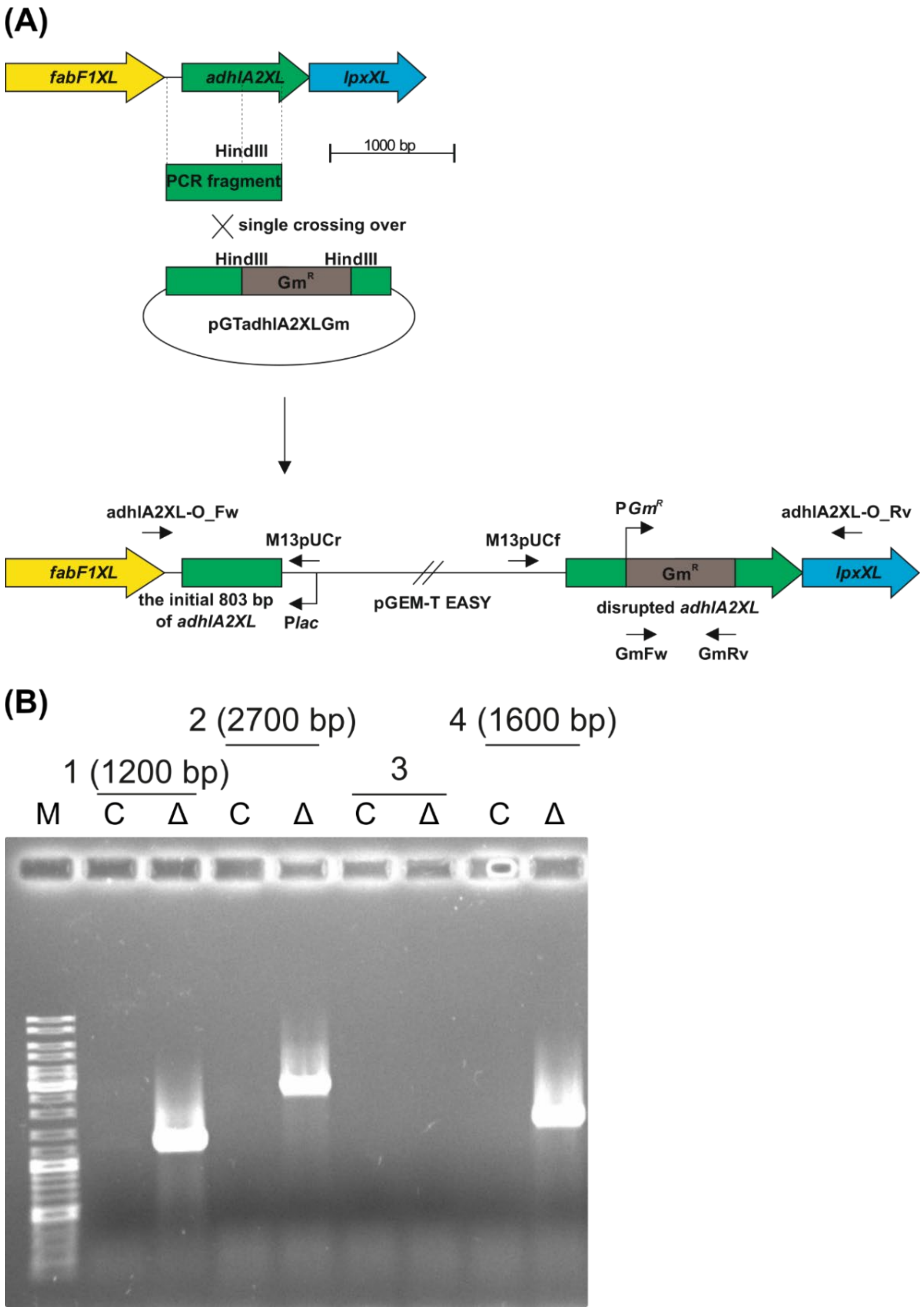

Supplementary Figure S2. Schematic representation of the steps involved in the construction of the *A. fabrum* C58 *adhlA22XL* insertional mutant (**A**) and the results of PCR confirming successful mutagenesis (**B**). The following primer pairs were used for DNA amplification: adhlA2-O_Fw and M13pUCr for fragment 1, M13pUCf and adhlA2XL-O_Fw for fragment 2, M13pUCf and GmFw for fragment 3, and M13pUCf and GmRv for fragment 4. The last two primer pairs were used to confirm the orientation of the gentamicin resistance cassette within *adhlA2XL*

.

Supplementary Table S5. Masses and proposed compositions of selected ions observed in MALDI-TOF MS of intact lipids A isolated from *A. fabrum* C58 and its mutants and complementants with examples of fragment ions.

No.	Observed ion (*m/z*)	Calculated monoisotopic mass	Molecular formula	Proposed composition	Additional comments
1	1996.3844	1996.4261	$C_{107}H_{205}N_2O_{26}P_2$	2 × GlcN;<br>1 × 16:0-(3OH)<br>1 × 18:0-(3OH)<br>2 × 14:0-(3OH)<br>1 × 28:0-(27OH)<br>1 × 4:0-(3OH)<br>2 × P	
2	1916.4271	1916.4261	$C_{106}H_{200}N_2O_{24}P_1$	2 × GlcN;<br>1 × 16:0-(3OH)<br>1 × 18:0-(3OH)<br>2 × 14:0-(3OH)<br>1 × 28:0-(27OH)<br>1 × 4:0-(3OH)<br>1 × P	
3	1914.4271	1914.4077	$C_{106}H_{198}N_2O_{24}P_1$	2 × GlcN;<br>1 × 16:0-(3OH)<br>1 × 18:1-(3OH)<br>2 × 14:0-(3OH)<br>1 × 28:0-(27OH)<br>1 × 4:0-(3OH)<br>1 × P	
4	1888.3972	1888.4285	$C_{105}H_{200}N_2O_{23}P_1$	2 × GlcN;<br>2 × 16:0-(3OH)<br>2 × 14:0-(3OH)<br>1 × 28:0-(27OH)<br>1 × 4:0-(3OH)<br>1 × P	
5	1886.3663	1886.3764	$C_{105}H_{198}N_2O_{23}P_1$	2 × GlcN;<br>1 × 16:0-(3OH)<br>1 × 16:1-(3OH)<br>2 × 14:0-(3OH)<br>1 × 28:0-(27OH)<br>1 × 4:0-(3OH)<br>1 × P	tr
6	1830.3951	1830.4230	$C_{103}H_{198}N_2O_{21}P_1$	2 × GlcN;<br>1 × 16:0-(3OH)<br>1 × 18:0-(3OH)<br>2 × 14:0-(3OH)<br>1 × 28:0-(27OH)<br>1 × P	
7	1802.3705	1802.3917	$C_{101}H_{194}N_2O_{21}P_1$	2 × GlcN;<br>2 × 16:0-(3OH)<br>2 × 14:0-(3OH)<br>1 × 28:0-(27OH)<br>1 × P	
8	1690.2371	1690.2301	$C_{92}H_{174}N_2O_{22}P_1$	2 × GlcN;<br>1 × 16:0-(3OH)<br>1 × 18:0-(3OH)<br>1 × 14:0-(3OH)<br>1 × 28:0-(27OH)<br>1 × 4:0-(3OH)<br>1 × P	

9	1604.1952	1604.1934	$C_{92}H_{174}N_2O_{22}P_1$	2 × GlcN<br>1 × 16:0-(3OH)<br>1 × 18:0-(3OH)<br>1 × 14:0-(3OH)<br>1 × 28:0-(27OH)<br>1 × P	
10	1662.2067	1662.1988	$C_{90}H_{170}N_2O_{22}P_1$	2 × GlcN<br>2 × 16:0-(3OH)<br>1 × 14:0-(3OH)<br>1 × 28:0-(27OH)<br>1 × 4:0-(3OH)<br>1 × P	
11	1576.1643	1576.1621	$C_{86}H_{164}N_2O_{20}P_1$	2 × GlcN<br>2 × 16:0-(3OH)<br>1 × 14:0-(3OH)<br>1 × 28:0-(27OH)<br>1 × P	
12	**1399.9930**	**1399.9844**	**$C_{76}H_{140}N_2O_{18}P_1$**	**2 × GlcN –H_2O**<br>**2 × 16:0-(3OH)**<br>**1 × 28:0-(27OH)**<br>**1 × 4:0-(3OH)**<br>**1 × P**	**fragment ion**
13	**1428.0199**	**1428.0157**	**$C_{78}H_{144}N_2O_{18}P_1$**	**2 × GlcN-H_2O**<br>**1 × 16:0-(3OH)**<br>**1 × 18:0-(3OH)**<br>**1 × 28:0-(27OH)**<br>**1 × 4:0-(3OH)**<br>**1 × P**	**fragment ion**
14	1464.1869	1464.0369	$C_{78}H_{148}N_2O_{20}P_1$	2 × GlcN<br>1 × 16:0-(3OH)<br>1 × 18:0-(3OH)<br>1 × 28:0-(27OH)<br>1 × 4:0-(3OH)<br>1 × P	
15	1774.1810	1774.16784	$C_{92}H_{172}N_2O_{24}P_2Na_1$	2 × GlcN<br>1 × 16:0-(3OH)<br>1 × 18:0-(3OH)<br>2 × 14:0-(3OH)<br>1 × 18:1<br>2 × P<br>1 × Na	
16	1750.1748	1752.1859	$C_{92}H_{172}N_2O_{24}P_2$	2 × GlcN<br>1 × 16:0-(3OH)<br>1 × 18:0-(3OH)<br>2 × 14:0-(3OH)<br>1 × 18:1<br>2 × P	
17	1672.2349	1672.2196	$C_{92}H_{172}N_2O_{21}P_1$	2 × GlcN<br>1 × 16:0-(3OH)<br>1 × 18:1-(3OH)<br>2 × 14:0-(3OH)<br>1 × 18:0<br>1 × P	2 × GlcN<br>1 × 16:0-(3OH)<br>1 × 18:0-(3OH)<br>2 × 14:0-(3OH)<br>1 × 18:1<br>1 × P
18	1670.2162	1670.2039	$C_{92}H_{170}N_2O_{21}P_1$	2 × GlcN<br>1 × 16:0-(3OH)<br>1 × 18:1-(3OH)<br>2 × 14:0-(3OH)<br>1 × 18:1<br>1 × P	

19	1644.1971	1644.18827	$C_{90}H_{168}N_2O_{21}P_1$	2 × GlcN<br>1 × 16:0-(3OH)<br>1 × 18:1-(3OH)<br>2 × 14:0-(3OH)<br>1 × 16:0<br>1 × P	2 × GlcN<br>1 × 16:0-(3-OH)<br>1 × 18:0-(3-OH)<br>2 × 14:0-(3-OH)<br>1 × 16:1<br>1 × P
20	1642.1782	1642.17262	$C_{90}H_{166}N_2O_{21}P_1$	2 × GlcN<br>1 × 16:0-(3OH)<br>1 × 18:1-(3OH)<br>2 × 14:0-(3OH)<br>1 × 16:1<br>1 × P	
21	1618.1890	1618.17267	$C_{88}H_{166}N_2O_{21}P_1$	2 × GlcN<br>2 × 16:0-(3OH)<br>2 × 14:0-(3OH)<br>1 × 16:0<br>1 × P	
22	1616.1687	1616.1569	$C_{88}H_{164}N_2O_{21}P_1$	2 × GlcN<br>2 × 16:0-(3OH)<br>2 × 14:0-(3OH)<br>1 × 16:1<br>1 × P	
23	1446.0386	1446.0262	$C_{78}H_{146}N_2O_{19}P_1$	2 × GlcN<br>1 × 16:0-(3OH)<br>1 × 18:0-(3OH)<br>1 × 14:0-(3OH)<br>1 × 18:0<br>1 × P	
24	1444.0292	1444.0106	$C_{78}H_{144}N_2O_{19}P_1$	2 × GlcN<br>1 × 16:0-(3OH)<br>1 × 18:1-(3OH)<br>1 × 14:0-(3OH)<br>1 × 18:1<br>1 × P	
25	1418.0000	1417.9949	$C_{76}H_{142}N_2O_{19}P_1$	2 × GlcN<br>2 × 16:0-(3OH)<br>1 × 14:0-(3OH)<br>1 × 18:1<br>1 × P	2 × GlcN<br>1 × 16:0-(3OH)<br>1 × 18:0-(3OH)<br>1× 14:0-(3OH)<br>1 × 16:1<br>1 × P
26	1399.993	1399.9844	$C_{76}H_{140}N_2O_{18}P_1$	2 × GlcN – H_2O<br>2 × 16:0-(3OH)<br>1 × 14:0-(3OH)<br>1 × 18:1<br>1 × P	fragment ion
27	1391.9792	1391.9793	$C_{74}H_{140}N_2O_{19}P_1$	2 × GlcN<br>2 × 16:0-(3OH)<br>1 × 14:0-(3OH)<br>1 × 16:0<br>1 × P	
28	1389.9752	1389.9636	$C_{74}H_{138}N_2O_{19}P_1$	2 × GlcN<br>2 × 16:0-(3OH)<br>1 × 14:0-(3OH)<br>1 × 16:1<br>1 × P	
29	1165.7150	1165.7860	$C_{60}H_{114}N_2O_{17}P_1$	2 × GlcN<br>2 × 16:0-(3OH)<br>1 × 16:0<br>1 × P	

30	1155.7834	1155.7806	$C_{62}H_{112}N_2O_{15}P_1$	2 × GlcN – 2×H_2O<br>2 × 16:0-(3OH)<br>1 × 18:1<br>1 × P	fragment ion

Above the green line: Ions characteristic for lipid A containing VLCFAs and their interpretation based on MS/MS spectra (data not shown). Below the green line: Ions characteristic for lipid A isolated from mutants (deprived of VLCFAs) and their interpretation based on MS/MS spectra.

A)

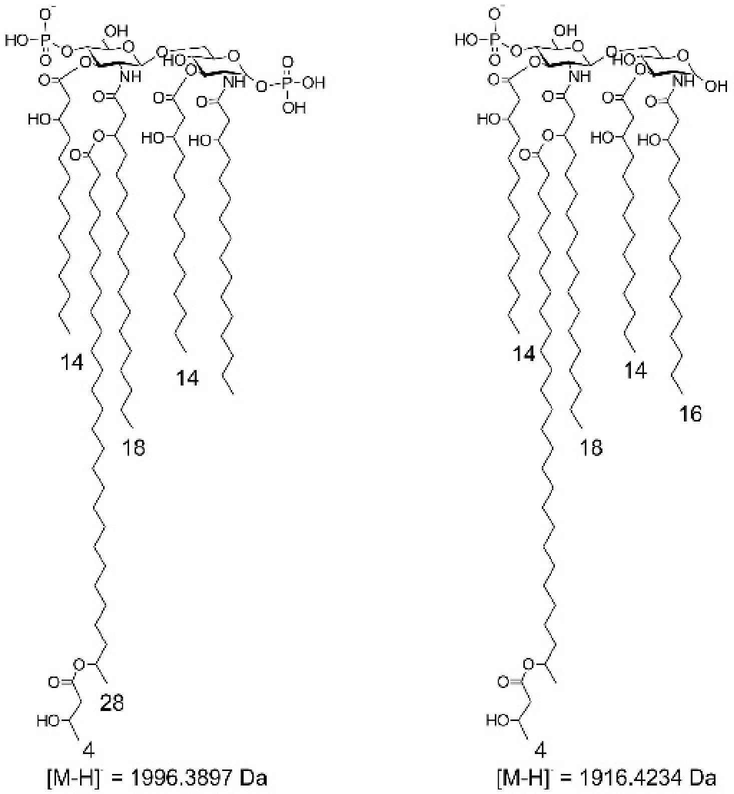

B)

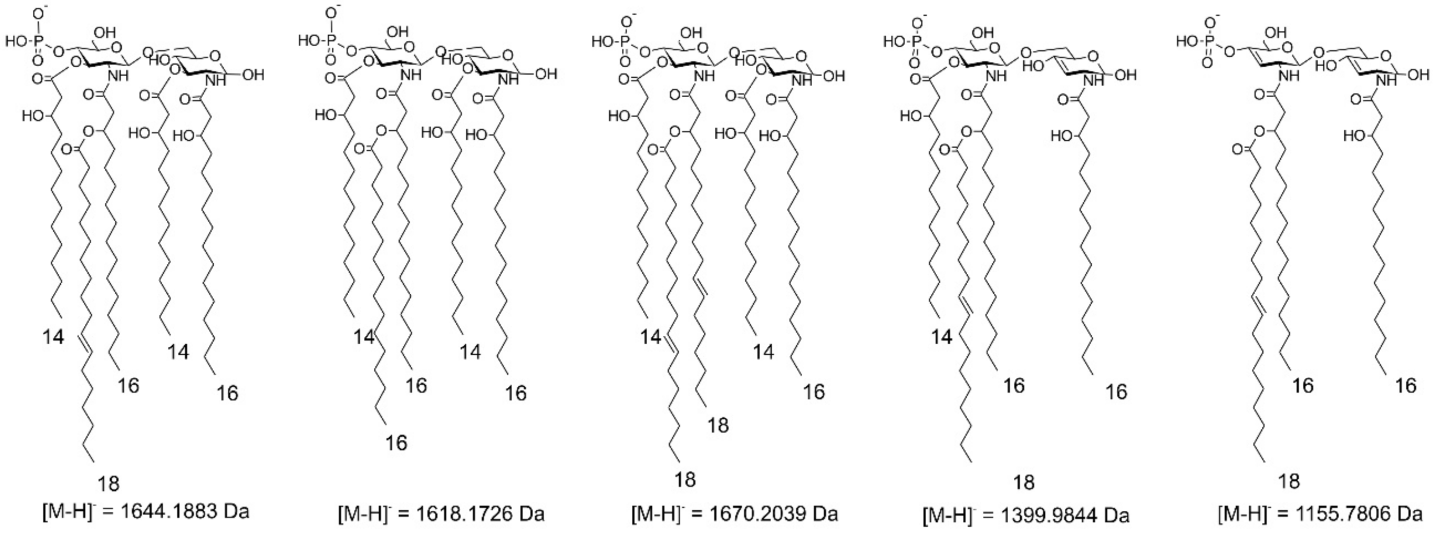

Supplementary Figure S3. Proposed structures of the main species of lipid A identified in: **A)** *A. fabrum* C58Wt and **B)** mutant strains deprived of VLCFAs and two examples of fragment ions with double bonds within sugar rings.

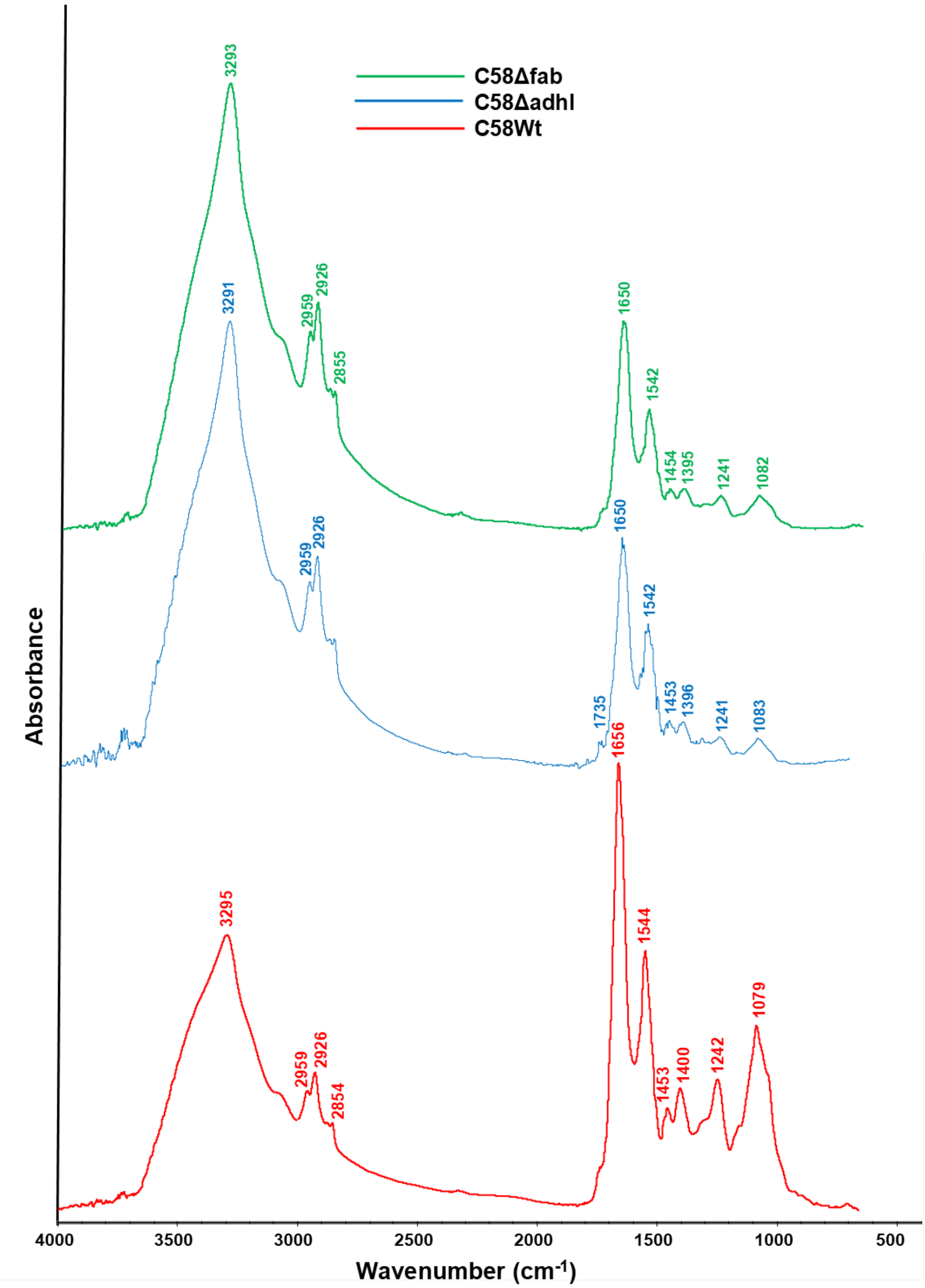

Figure S4. Representative FTIR spectra of biofilms formed at the abiotic surface by *A. fabrum* C58Wt (red line) and mutants C58Δadhl (blue line) and C58Δfab (green line).

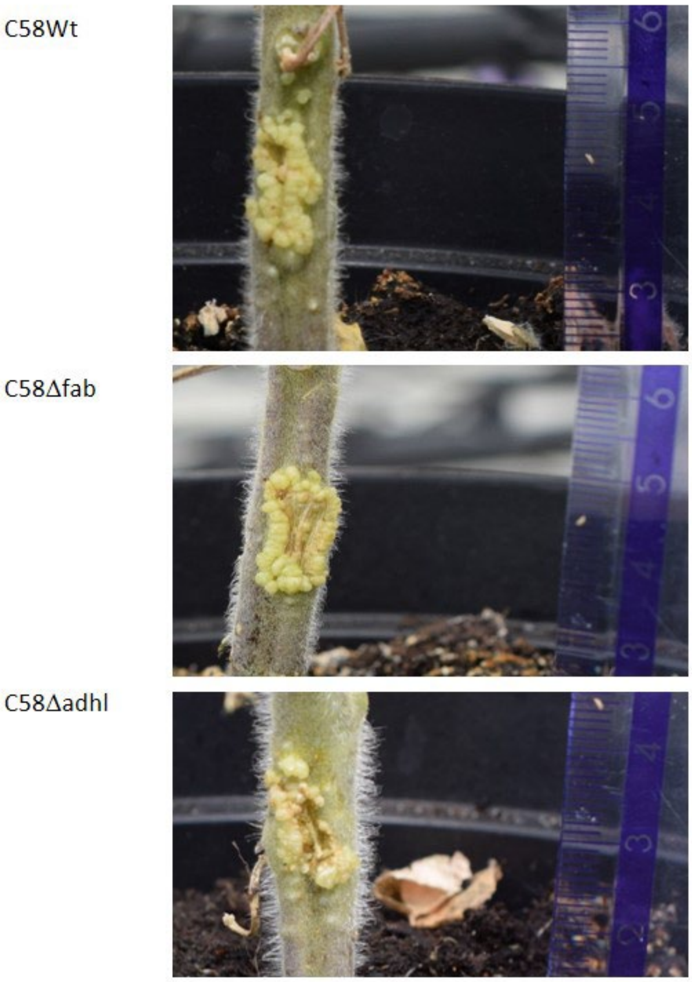

Supplementary Figure S5. Tomato seedlings infected with *A. fabrum* C58Wt and mutants C58Δfab and C58Δadhl. Plant stems were photographed 6 weeks after infection. A ruler placed next to the seedling allows estimating the size of changes on the tomato stems.

Supplementary Table S6. Composition of the bacterial media used in this study.

Component	Concentration [g × L^{-1}]		
	79CA	M1	TY
**Mannitol** [1]	10.0	10.0	10.0
**Yeast extract** [2]	1.0	–	3.0
**Acid hydrolyzed casein** [3]	1.0	–	–
**Calcium glycerophosphate** [4]	0.1	–	–
**Tryptone** [4]	–	–	5.0
**$CaCl_2 \times 7H_2O$** [5]	–	–	1.3
**K_2HPO_4** [5]	0.5	2.0	–
**NaCl** [5]	0.1	0.1	–
**$MgSO_4 \times 7H_2O$** [5]	0.2	0.2	–
**NH_4Cl** [5]	–	1.0	–
**KH_2PO_4** [5]	–	0.5	–

[1] Chempur, Piekary Śląskie, Poland

[2] A&A Biotechnology, Gdańsk, Poland

[3] Thermo Fisher Scientific, Waltham, MA, USA

[4] Merck KGaA, Darmstadt, Germany

[5] Avantor Performance Materials, Gliwice, Poland

Supplementary References:

1. Żebracki, K.; Koper, P.; Marczak, M.; Skorupska, A.; Mazur, A. Plasmid-Encoded RepA Proteins Specifically Autorepress Individual *repABC* Operons in the Multipartite *Rhizobium Leguminosarum* Bv. *Trifolii* Genome. *PLoS One* **2015**, *10*, e0131907, doi:10.1371/journal.pone.0131907.

2. Cervantes-Rivera, R.; Pedraza-López, F.; Pérez-Segura, G.; Cevallos, M.A. The Replication Origin of a *repABC* Plasmid. *BMC Microbiol* **2011**, *11*, 158, doi:10.1186/1471-2180-11-158.

3. Kovach, M.E.; Elzer, P.H.; Hill, D.S.; Robertson, G.T.; Farris, M.A.; Roop, R.M.; Peterson, K.M. Four New Derivatives of the Broad-Host-Range Cloning Vector pBBR1MCS, Carrying Different Antibiotic-Resistance Cassettes. *Gene* **1995**, *166*, 175–176, doi:10.1016/0378-1119(95)00584-1.
